# Coordination Cage‐Based Emulsifiers: Templated Formation of Metal Oxide Microcapsules Monitored by In Situ LC‐TEM

**DOI:** 10.1002/chem.202103406

**Published:** 2021-12-21

**Authors:** Subhadeep Saha, Yen‐Ting Chen, Sudhakar Ganta, Markus Gilles, Björn Holzapfel, Pascal Lill, Heinz Rehage, Christos Gatsogiannis, Guido H. Clever

**Affiliations:** ^1^ Department of Chemistry and Chemical Biology TU Dortmund University Otto-Hahn Straße 6 44227 Dortmund Germany; ^2^ Center of Molecular Spectroscopy and Simulation of Solvent-driven Processes (ZEMOS) Ruhr-University, Bochum 44801 Bochum Germany; ^3^ Department of Structural Biochemistry Max Planck Institute of Molecular Physiology Otto-Hahn Straße 11 44227 Dortmund Germany; ^4^ Institute for Medical Physics and Biophysics and Center for Soft Nanoscience Westfälische Wilhelms-University Münster Busso-Peus Str. 10 48149 Münster Germany

**Keywords:** coordination cages, emulsions, metal oxides, self-assembly, transmission electron microscopy

## Abstract

Metallo‐supramolecular self‐assembly has yielded a plethora of discrete nanosystems, many of which show competence in capturing guests and catalyzing chemical reactions. However, the potential of low‐molecular bottom‐up self‐assemblies in the development of structured inorganic materials has rarely been methodically explored so far. Herein, we present a new type of metallo‐supramolecular surfactant with the ability to stabilize non‐aqueous emulsions for a significant period. The molecular design of the surfactant is based on a heteroleptic coordination cage (**CGA‐3**; **CGA**=Cage‐based Gemini Amphiphile), assembled from two pairs of organic building blocks, grouped around two Pd(II) cations. Shape‐complementarity between the differently functionalized components generates discrete amphiphiles with a tailor‐made polarity profile, able to stabilize non‐aqueous emulsions, such as hexadecane‐in‐DMSO. These emulsions were used as a medium for the synthesis of spherical metal oxide microcapsules (titanium oxide, zirconium oxide, and niobium oxide) from soluble, water‐sensitive alkoxide precursors by allowing a controlled dosage of water to the liquid‐liquid phase boundary. Synthesized materials were analyzed by a combination of electron microscopic techniques. In situ liquid cell transmission electron microscopy (LC‐TEM) was utilized for the first time to visualize the dynamics of the emulsion‐templated formation of hollow inorganic titanium oxide and zirconium oxide microspheres.

## Graphical abstract

**Self‐assembled amphiphiles captured in action**: Non‐statistical self‐assembly of heteroleptic coordination cages results in Gemini amphiphiles, able to stabilize non‐aqueous emulsions. Serving as water‐free medium, the emulsion‐templated synthesis of metal oxide microcapsules from water‐sensitive precursors can be carried out in controlled fashion. The formation of hollow metal oxide microcapsules was visualized by in situ liquid cell transmission electron microscopy and elemental mapping.

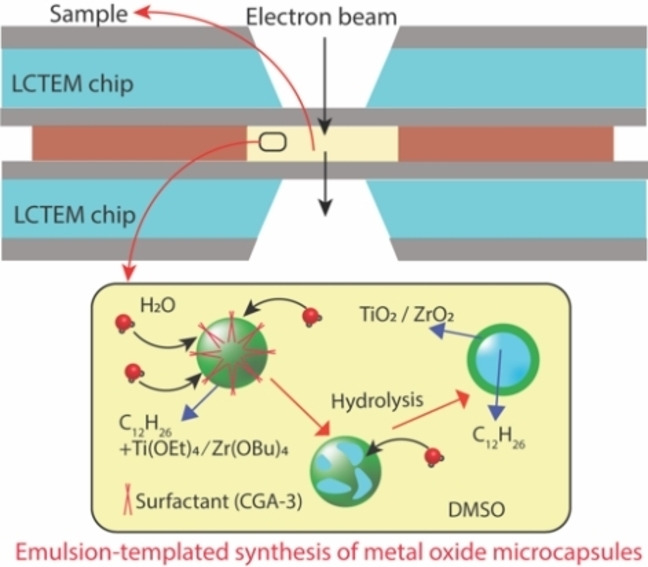

## Introduction

Supramolecular self‐assembly is one of the most efficient processes to create functional nanostructures from carefully engineered molecular components.[^1^, ^2^, ^3^, ^4^, ^5^, ^6^, ^7^, ^8^, ^9^] Functional multicomponent systems are ubiquitous in nature; for example multimeric enzyme complexes, which utilize their microenvironment and reactive interior to promote reactions with remarkable rate acceleration, substrate specificity, and product selectivity.^[10]^ During the past four decades, the development of rational self‐assembly approaches resulted in a large variety of artificial hosts of nanometre dimensions with applications in selective recognition, adaptive guest binding, stabilization of reactive intermediates, and catalysis.[^11^, ^12^, ^13^, ^14^, ^15^, ^16^, ^17^, ^18^] However, the potential of such discrete self‐assemblies as tools in the development of structured inorganic materials has rarely been tested.[^19^, ^20^]

Surfactants or amphiphiles consist of two different moieties, one with hydrophilic/polar and the second with hydrophobic/non‐polar character, connected via covalent and dynamic covalent bonding, as well as noncovalent interactions.[^21^, ^22^, ^23^, ^24^, ^25^] The latter strategy opens the possibility to combine polar and non‐polar components in a modular self‐assembly approach. Recently, our group has reported a multicomponent coordination cage (**CGA‐1**; section 1.2, Supporting Information) of anisotropic shape, comprising a polar head unit (four methoxy groups close to two cationic Pd(II) centers) and non‐polar dodecyl appendices, capable of stabilizing non‐aqueous emulsions.^[26]^ Herein, we report the assembly of an optimized derivative (**CGA‐3**), possessing considerable solubility in both polar (DMSO, acetonitrile, DMF, etc.) as well as non‐polar solvents (hexadecane, octane, toluene, etc.), and featuring a much improved efficiency in stabilizing non‐aqueous emulsions. It has often been observed that high‐molecular‐weight compounds (polymeric surfactants)^[27]^ and solid particles^[28]^ perform superior as emulsifiers for a mixture of two immiscible organic liquids compared to conventional low‐molecular surfactants, as the latter seldomly possess sufficient solubility in both of the phases. **CGA‐3** is a first‐of‐its‐kind discrete supramolecular surfactant which can efficiently stabilize the emulsion of hexadecane‐in‐DMSO (DMSO:hexadecane=4 : 1) for a significant period of time (∼50 days; Figure S20) at a concentration as low as 0.2 mM. Non‐aqueous emulsions have a broad range of applications,^[29]^ including drug delivery,[^30^, ^31^, ^32^] as platform for emulsion polymerization reactions[^27^, ^33^, ^34^, ^35^] and for the emulsion‐templated syntheses of hollow inorganic materials (e. g. porous inorganic oxides).[^36^, ^37^, ^38^, ^39^, ^40^, ^41^]

In the past couple of decades, scientists have made remarkable progress in the development of functional porous inorganic oxide materials for application in several domains of technology, for example as micro‐/nanocontainers and reactors, optical and magnetic materials, energy storage components, photocatalysts, sensors as well as in biomedical and environmental remediation applications.[^36^, ^37^] Hence, the elaboration of efficient methods for the synthesis of porous inorganic oxides has become essential. Non‐aqueous, emulsion‐templated approaches, based on the controlled reaction of hydrolytically unstable precursors (e. g. tetraethyl orthosilicate, alkoxides of Ti(IV), Zr(IV), Nb(V), etc.) promise performance and applicability in this context. However, examples of small‐molecule surfactant‐stabilized non‐aqueous emulsions as a medium for the synthesis of metal oxide capsules are rare. Herein, we show that supramolecular amphiphile **CGA‐3** stabilizes emulsions composed of two immiscible organic liquids (hexadecane‐in‐DMSO) and can be used as a medium to synthesize inorganic oxide microcapsules (titanium oxide, zirconium oxide, niobium oxide) at room temperature.

The formation of solid materials from a solution phase is an ubiquitous phenomenon in biological, geochemical and synthetic systems. Analysis of the underlying processes by spatially and temporally resolving imaging techniques, however, is challenging. The recent advancements of liquid cell transmission electron microscopy (LC‐TEM) are transforming our understanding of the physical and chemical mechanisms controlling the formation of materials from solution.[^42^, ^43^, ^44^, ^45^, ^46^, ^47^, ^48^, ^49^, ^50^] Emulsions have been known for decades as a medium of synthesis for a large variety of materials. While mechanisms underlying the emulsion‐templated synthesis of micro‐structured solid phases have been predicted,[^38^, ^39^] there has been little progress to visualize such processes by high‐resolution microscopic techniques. Herein, we give insight into the dynamics of the hydrolytic formation of titanium and zirconium oxide microspheres from hexadecane‐in‐DMSO emulsions, using time‐resolved in situ transmission electron microscopy (TEM) techniques.

## Results and Discussion

**Figure 1:**
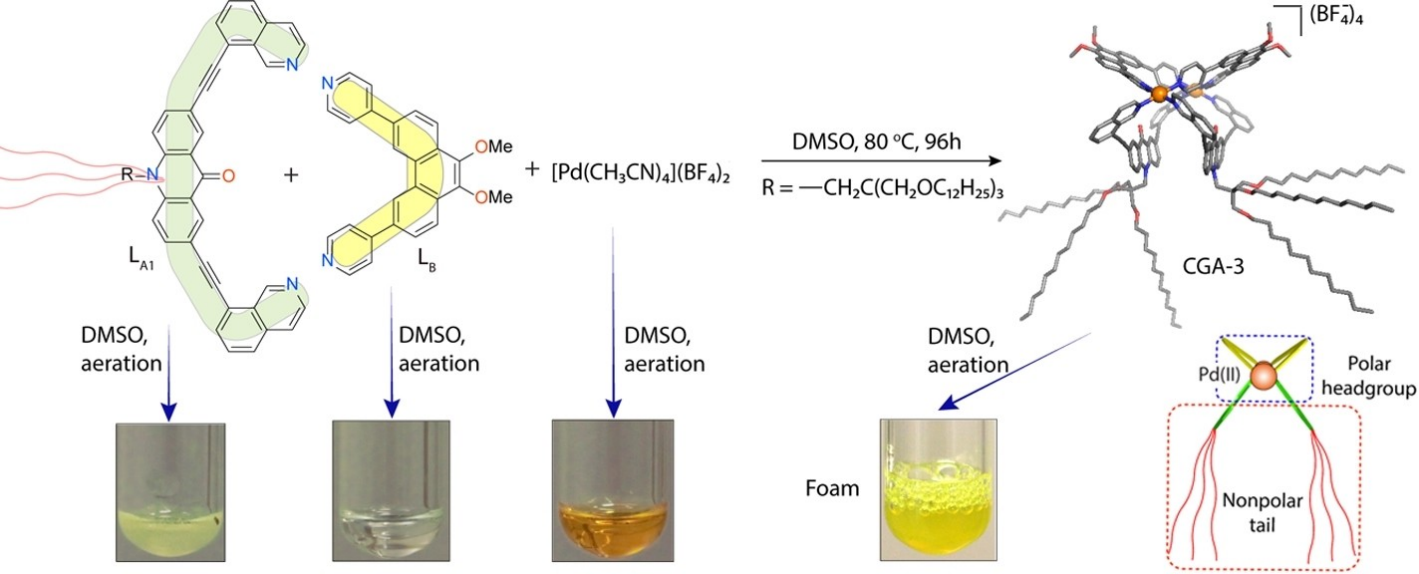
Shape‐complementary assembly of supramolecular surfactant **CGA‐3** (**C**age‐based **G**emini **A**mphiphile‐3), its PM6‐optimized structure and foaming capabilities of individual components and the cage compared.

The design of **CGA‐3** was inspired by a first‐generation amphiphilic cage (**CGA‐1**), based on a heteroleptic assembly scheme and recently reported by our group.^[26]^ The structure of **CGA‐3** is generated by the shape‐complementarity of ligands **L^A1^** and **L^B^**,[^51^, ^52^] carrying non‐polar and polar substituents, respectively. Integrative self‐sorting[^4^, ^52^, ^53^] of **L^A1^** and **L^B^** with [Pd(CH_3_CN)_4_](BF_4_)_2_ in a 1 : 1 : 1 ratio in DMSO leads to the formation of **CGA‐3** (=*cis*‐[Pd_2_(**L^A1^**)_2_(**L^B^**)_2_]^4+^) as the thermodynamic product (Figure 1; for detailed synthetic procedures see the Supporting Information). It is worth mentioning that **L^A1^** and **L^B^** are capable of separately reacting with the palladium precursor [Pd(CH_3_CN)_4_](BF_4_)_2_ to form rather non‐polar homoleptic cage [Pd_2_(**L^A1^**)_4_]^4+^ (C1) and highly polar ring [Pd_4_(**L^B^**)_8_]^8+^ (C2),^[51]^ respectively (Section 1.4, Supporting Information). In combination, however, the perfectly matching contours of the two ligands allow modular formation of a single heteroleptic product instead of leading to narcissistic self‐sorting or statistical mixtures. The **CGA‐3** complex was characterized by high‐resolution electrospray‐ionization time‐of‐flight mass spectrometry (HR‐ESI‐TOF‐MS; Figure S11), showing the presence of two dominant species with mass/charge ratio (*m/z*) corresponding to [Pd_2_(**L^A1^**)_2_(**L^B^**)_2_(BF_4_)]^3+^ and [Pd_2_(**L^A1^**)_2_(**L^B^**)_2_]^4+^. DFT calculations on a related heteroleptic cage with analogous core structure indicated that the formation of the *cis*‐configured isomer of [Pd_2_(**L^A1^**)_2_(**L^B^**)_2_] (Figure 1) is energetically more favorable than a tentative *trans*‐configuration.^[51]^

**Figure 2:**
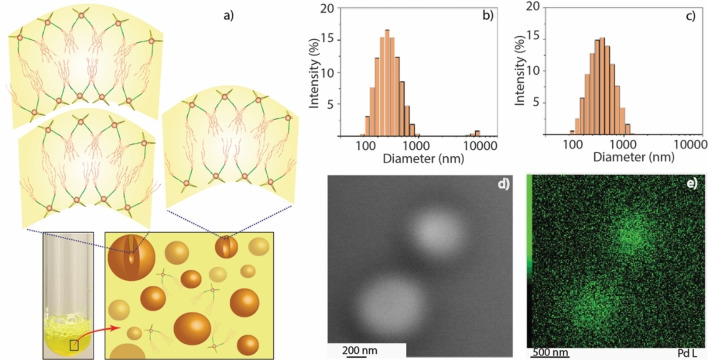
a) Cartoon representation of surfactant (**CGA‐3**), aggregating to form vesicles in DMSO; b–c) DLS data of solutions of **CGA‐3** (0.1 mM and 0.2 mM, respectively); d) HAADF‐STEM (liquid phase) images of vesicles (200–500 nm) in DMSO (0.2 mM; sample A); e) EDX elemental intensity maps (liquid phase) confirming the vesicles are made up of palladium‐based surfactants (**CGA‐3**).

The structure of **CGA‐3** resembles a Gemini amphiphile as it is made up of two identical amphiphilic units (Figure 1), connected by the two metal nodes. Gemini amphiphiles can exhibit surface activities in water at low concentration which is the reason behind their vast applicability in solubilization, separation and transportation processes as well as in catalytic reactions.[^54^, ^55^] At ambient temperature, **CGA‐3** forms a translucent colloidal suspension (0.2 mM, Figure 1) in polar organic solvents, for example DMSO or acetonitrile. Formation of a foam (stable for ∼3 h at ambient atmosphere) upon passage of air through the DMSO solution indicates that **CGA‐3** is a potential surfactant for polar aprotic solvents like DMSO. It is noteworthy that previously reported **CGA‐1** does not show the formation of foams under similar experimental conditions.^[26]^ Dynamic light scattering (DLS) studies of the **CGA‐3** solutions in DMSO at two different concentrations (0.2 mM; sample A and 0.1 mM; sample B; Figure S13) indicate the presence of well‐dispersed colloidal particles with various hydrodynamic diameters (200–500 nm) under ambient conditions.^[26]^ To gain further insight into the shape and nature of the particles, we performed electron microscopy under cryogenic conditions (cryo‐TEM) and LC‐TEM experiments^[41]^ which are established methods for analyzing particles in vitrified liquids at low temperature (80 K) and in the liquid state at ambient conditions, respectively (Figure 2, S14 and S15).^[42]^ Cryo‐TEM analyses of sample A showcased the presence of spherical particles with diameters of 300–400 nm (Figure S14). In situ LC‐TEM analysis of sample A also shows the presence of polydispersed vesicles with diameters of 300–400 nm (Figure 2, S15), which corroborates with the DLS data as well as the information obtained from cryo‐TEM analyses.

**Figure 3:**
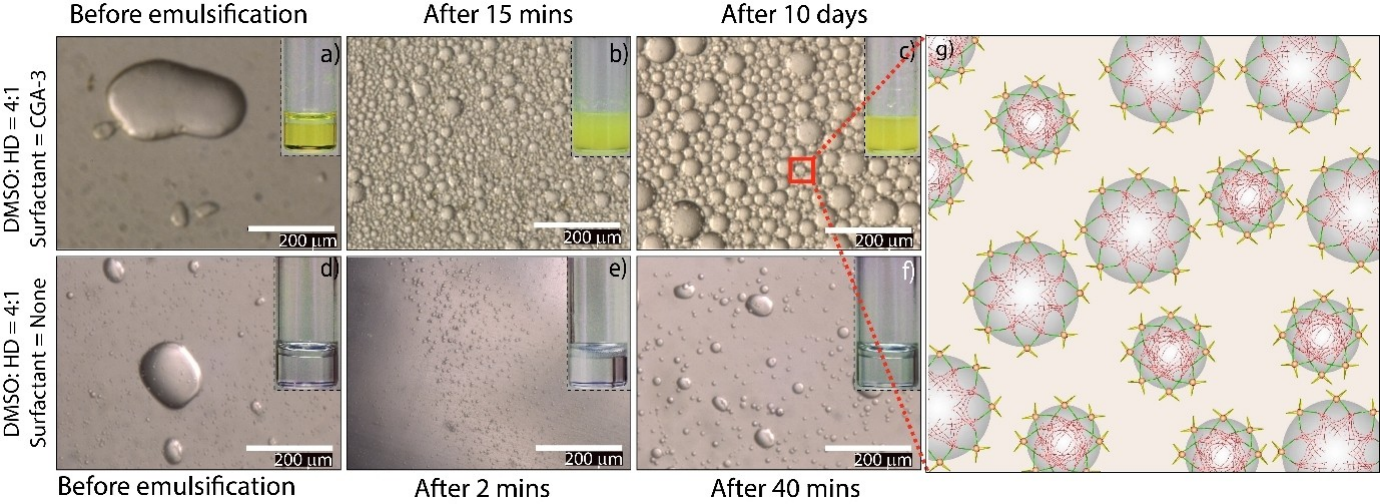
a) and d) Optical microscopy (OM) images of mixtures of DMSO and hexadecane (4 : 1) in presence (0.2 mM) and absence of **CGA‐3** surfactant, respectively; panels b) and c) show OM images of stable emulsions of mixtures containing **CGA‐3** upon homogenization; panels e) and f) indicate no formation of emulsions in mixtures without any surfactant, which is also clearly perceived from the macroscopic separation of the liquid phases (inset images). The cartoon image represents the formation of emulsion droplets (hexadecane) in a continuous phase (DMSO) by **CGA‐3**.

The high surface activity of **CGA‐3** in DMSO motivated us to examine its ability to act as an emulsifier. Notably, **CGA‐3** was found to be incapable of stabilizing any water‐based emulsions as it is virtually insoluble in water. Interestingly, **CGA‐3** was observed to efficiently stabilize a mixture of DMSO and hexadecane (ratio=4 : 1; concentration of **CGA‐3**=0.2 mM) to form emulsions (droplet size=10–50 μm; Figure 3) which remain stable for ∼50 days (Figure S20). In contrast, **CGA‐1** fails to stabilize a similar mixture of DMSO and hexadecane to form an emulsion. Notably, **CGA‐1** only transiently (<24 h) stabilizes a similar emulsion (acetonitrile : hexadecane=4 : 1, the concentration of **CGA‐1**: 0.2 mM), which unequivocally establishes the superior surface activity of **CGA‐3** compared to that of **CGA‐1**. This apparent disparity in surface activity can be attributed to the improved solubility of **CGA‐3** in non‐polar phases (Figure S16) due to the presence of more alkyl chains in **CGA‐3**. To the best of our knowledge, **CGA‐3** is a first‐of‐its‐kind supramolecular emulsifier, capable of stabilizing a non‐aqueous emulsion which can be utilized as a template for synthesizing metal oxide microcapsules from water‐sensitive precursors (next section).

**Figure 4:**
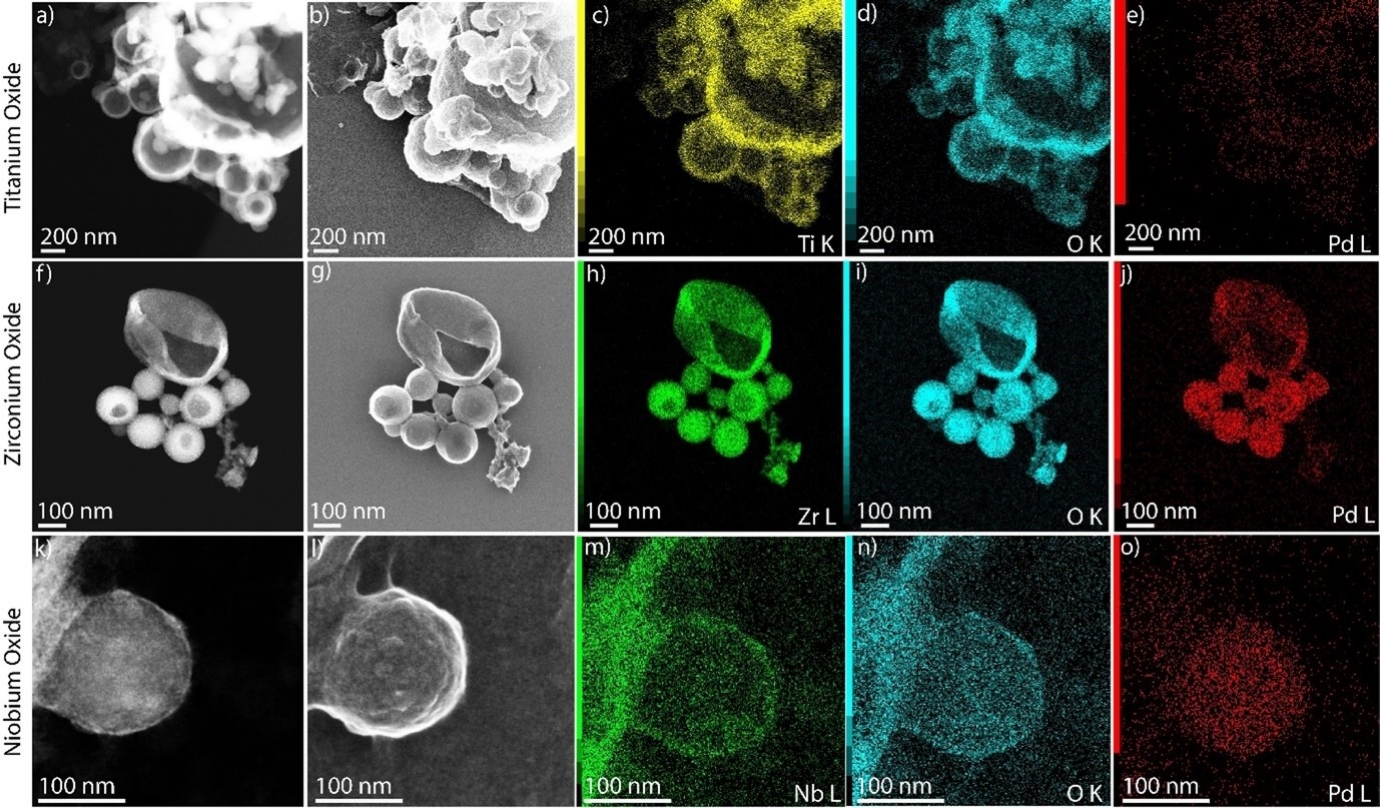
TEM analysis of the dried metal oxide microcapsules (titanium oxide, zirconium oxide, and niobium oxide) obtained by means of emulsion‐templated synthesis. a), f) and k) are HAADF‐STEM images and b), g), l) are SEI (secondary electron imaging) images of the microcapsules, respectively; c–e, h–j and m–o) EDX elemental intensity maps of the metal oxide microspheres (titanium oxide, zirconium oxide, and niobium oxide, respectively).

The superior performance of **CGA‐3** as an emulsifier for immiscible non‐aqueous solvent systems encouraged us to utilize such emulsions as a template for the synthesis of metal oxide microcapsules (titanium oxide, zirconium oxide, niobium oxide). First of all, it was interesting to notice that these emulsions (continuous phase=dry DMSO; dispersed phase=alkoxide precursor in dry hexadecane; surfactant=**CGA‐3**; section 7 in Supporting Information) were found to contain smaller droplets (100–500 nm; Figure S17) compared to the ones devoid of the alkoxide precursors (droplet size=10–50 μm; Figure 3), probably caused by an additional drop in interfacial tension. Spherical metal oxide microcapsules of different dimensions (Figure 4 and S18) were formed spontaneously upon introduction of minuscule amounts of water (10 μl/1 ml DMSO solution) to these emulsion systems (section 7 in Supporting Information) at ambient temperature (Figure S18).

After completion of the hydrolysis reaction, the microcapsules separate as solid floc (Figure S21b) which was collected by successive washing and centrifugation steps. The formation of hollow metal oxide microcapsules was confirmed by electron microscopy (TEM and High‐Angle Annular Dark‐Field Scanning Transmission Electron Microscopy, HAADF‐STEM) studies of the obtained solids, showing the formation of capsules of 100–1500 nm size (titanium oxide: 100–1500 nm; zirconium oxide: 100–500 nm; niobium oxide: 200–300 nm; Figure 4 and S18). Although TiO_2_ and ZrO_2_ particles are clearly visualized as hallow capsules, Nb_2_O_5_ particles are imaged as inorganic spherical particles. The reactivity and nature of metal precursors probably play a crucial role in the diffusion of the reagents. In a bid to ascertain the elemental composition of the capsules, the samples were analyzed using HAADF‐STEM in combination with Energy Dispersive Spectroscopy (EDS) which clearly showed the uniform distribution of the expected elements (Ti, Zr, and Nb for titanium oxide, zirconium oxide, and niobium oxide, respectively), S (from DMSO), O, Pd, N (from **CGA‐3**) and F (from BF_4_^–^ counter anions; Figure 4 and S18b) within the particles.

Next, the emulsion‐templated growth of the amorphous metal oxide microcapsules was examined by performing the reactions in situ, monitored by LC‐TEM. In order to obtain the optimum contrast at a limited flux of electrons, we used a beam current of 0.7 nA and electron flux of 1.1 e^−^Å ^−2^ per second (for titanium oxide: TEM mode) and 7 e^−^ Å^−2^ per second (for zirconium oxide: STEM‐Bright Field mode). No visible damage was observed under these electron beam conditions. Experimental details of the in situ experiments are described in section 10 of Supporting Information. Real‐time imaging of the reaction revealed the conversion of dense spherical droplets into hollow appearing ones in the presence of a water source. For these in situ experiments, two different solutions were prepared; solution A: a mixture of water and DMSO (1 μl water in 1 ml dry DMSO) and solution B: the emulsion containing alkoxide precursor (Ti(OEt)_4_ or Zr(OBu)_4_; section 7 in Supporting Information) in the dispersed phase. 1 μl of solution B was filled in an empty sample compartment of the liquid cell setup. Solution A (flow rate: 1 μl/min) was injected through a long tube for ∼15 minutes (including a dead‐time of a few minutes before it reached the sample) in order to carry out the hydrolysis reaction. After 15 min, most of the starting material was converted into solid oxides, indicated by minimal changes observable under the electron microscope (concerning the solution and formed spherical particles). In addition, the expelled liquid from the cell (collected in an Eppendorf® tube connected to an outlet pipe) shows the presence of white solids, attributed to excess metal oxide leaving the sample compartment.

On a microscopic level, we propose that right after water hits the surface of a dispersed oil droplet (i. e. Ti(OEt)_4_ dissolved in hexadecane), hydrolysis of the metal alkoxide takes place and titanium oxide forms as a separate, solid phase at the interface. This reaction triggers the build‐up of a concentration gradient of Ti(OEt)_4_ within the droplet, driving it from the bulk interior to the depleted interface. Hydrolysis of all Ti(OEt)_4_ ultimately forms the solid titanium oxide shell, which is evident from the real‐time in situ imaging results. The recorded data shows that after introduction of water, dark spots appear on the initially homogeneous white droplets (hexadecane phase containing alkoxide precursors), followed by enlargement of these spots with time, which we denote to the migration of the inorganic oxide precursor from the bulk to the surface of the droplet. Hollow spherical particles then form by solidification along the amphiphile‐stabilized surfaces to yield a continuous shell of metal oxide. On average, this conversion process takes about 10–12 seconds to complete for the droplets formed under the given conditions (Figure S19 and Supporting Movies). The morphology of the resulting spherical particles corroborates to that of the dried metal oxide microspheres obtained at larger scale as described above (Figure 4 and S18).

**Figure 5:**
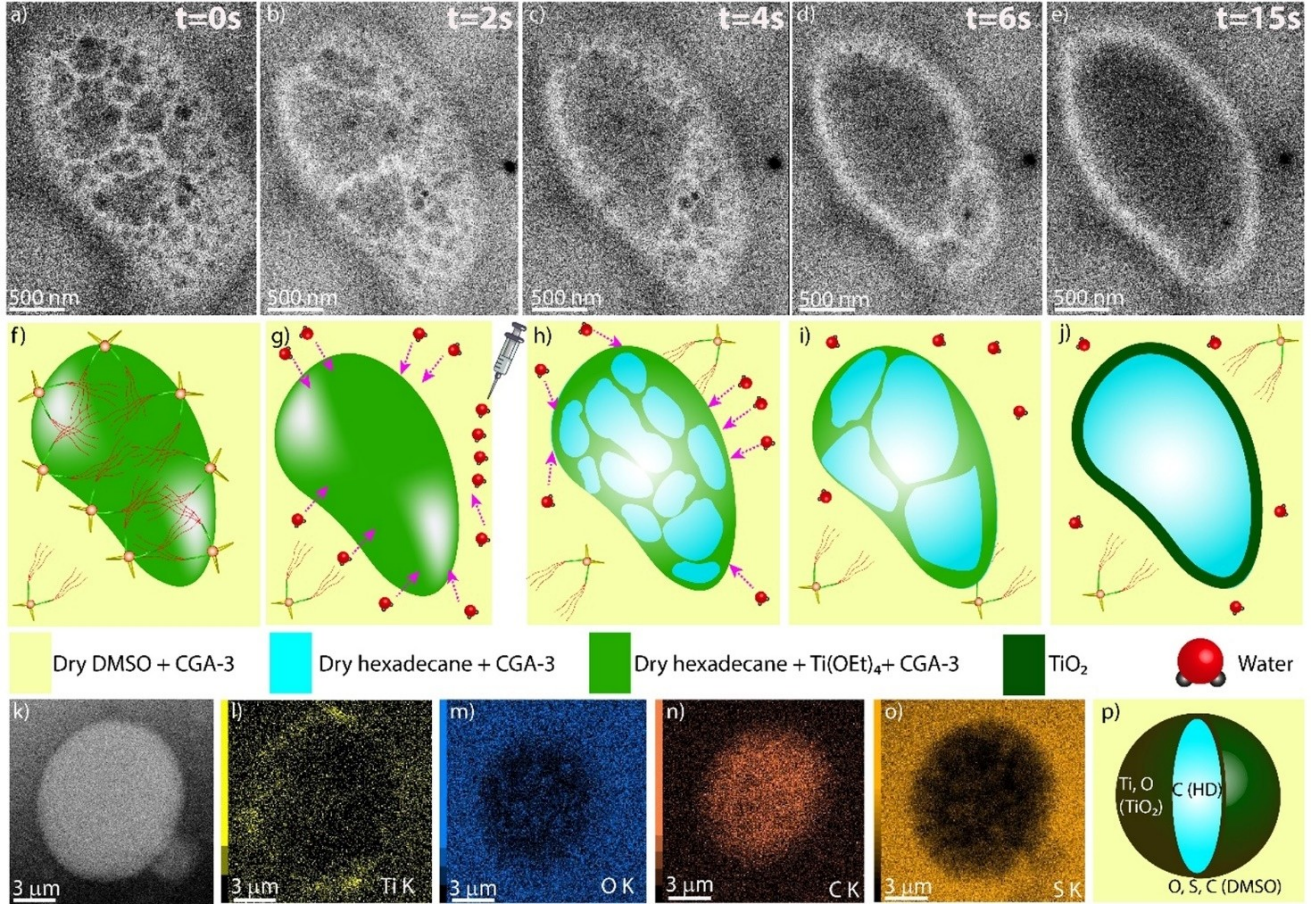
(a–e) Consecutive series of in situ LC‐TEM images showing the growth of a titanium oxide microsphere at the interface of dispersed (hexadecane) and continuous phase (DMSO) over time; f) cartoon representation of an emulsion droplet containing Ti(OEt)_4_ in the bulk phase and **CGA‐3** at the interface; g–j) growth of titanium oxide microspheres in the emulsion; k) HAADF‐STEM image and l–o) EDX elemental intensity maps of an as‐synthesized titanium oxide microcapsule during the in situ LC‐TEM experiment, p) combined information obtained from the EDX analysis (inner view showing presence of hexadecane HD).

To gain further information about the structure and elemental composition of the as‐synthesized titanium oxide microcapsules in the emulsion, the obtained suspension was analyzed by HAADF‐STEM, liquid state and EDS. This study revealed the existence of Ti only at the periphery of the spheres (interface of oil droplet and DMSO), hence supporting the hollow nature of the solid particles (Figure 5l). While the oxygen content of the particles cannot be distinguished from the oxygen content of the surrounding continuous phase (DMSO; Figure 5m), the carbon‐abundant (Figure 5n) and sulfur‐deficient (Figure 5o) core of the spheres (compared to the exterior DMSO phase) confirms the presence of hydrocarbon (hexadecane) as well as the absence of DMSO at the interior of the sphere. Thus, all these evidences direct towards the fact that the hollow titanium oxide spheres form on the hexadecane droplet templates, immersed in the DMSO phase, in the presence of water.

## Conclusion

In conclusion, we have utilized the potential of rational supramolecular self‐assembly to create a Gemini amphiphilic multicomponent coordination cage. This integrative self‐sorting process is driven by the shape‐complementarity of two differently functionalized ligands. The obtained supramolecular surfactant was found to be capable of decreasing the surface tension of DMSO significantly, leading to the formation of foams and vesicles. Interestingly, its efficacy as a surfactant is manifested in its ability to stabilize non‐aqueous emulsions at very low concentration, unlike conventional molecular amphiphiles. The non‐statistical modular character of this compound opens potential for the tailored development and rapid optimization of efficient alternatives to polymeric surfactants for non‐aqueous systems. Moreover, we could utilize the dispersed phase of these emulsions as template to synthesize spherical metal oxide microcapsules of Ti, Zr and Nb. As a certain amount of the Pd(II)‐based surfactant (**CGA‐3**) is expected to remain embedded in the formed metal oxide matrix, we envision this approach to allow generating single‐atom catalysts (herein Pd) after subsequent reductive treatment. Furthermore, in situ LC‐TEM was applied to visualize the formation dynamics of the solid oxide materials from the solution phase. EDS elemental intensity mapping during these in situ experiments unequivocally indicated formation of the metal oxide shells at the interface of the dispersed (hexadecane) and continuous phases (DMSO). The herein reported combination of an emulsion‐templated synthetic approach based on a novel supramolecular amphiphile with in situ electron microscopy imaging holds potential to spur further development at the intersection of bottom‐up self‐assembly of nanostructures with the field of emulsion‐based materials chemistry.

## Experimental Section

The detailed synthesis and characterization of all the compounds, DLS, emulsification experiments, TEM, and LC‐TEM methods are described in the Supporting Information.

## Conflict of interest

The authors declare no conflict of interest.

## Supporting information

As a service to our authors and readers, this journal provides supporting information supplied by the authors. Such materials are peer reviewed and may be re‐organized for online delivery, but are not copy‐edited or typeset. Technical support issues arising from supporting information (other than missing files) should be addressed to the authors.

Supporting Information

Supporting Information
