# Supplementary material for: Coordination Cage‐Based Emulsifiers: Templated Formation of Metal Oxide Microcapsules Monitored by In Situ LC‐TEM
Source: Chemistry. 2021 Dec 21;28(5):e202103406. doi: 10.1002/chem.202103406 (PMC9299919; doi:10.1002/chem.202103406)
Supplement: Supplementary file 1 — Supporting Information [file CHEM-28-0-s002.pdf]

Chemistry–A European Journal

Supporting Information

Coordination Cage-Based Emulsifiers: Templated Formation of Metal Oxide Microcapsules Monitored by In Situ LC-TEM

Subhadeep Saha, Yen-Ting Chen, Sudhakar Ganta, Markus Gilles, Björn Holzapfel, Pascal Lill, Heinz Rehage, Christos Gatsogiannis, and Guido H. Clever*

Supporting Information

Contents

1. Experimental Section 2

1.1. Materials and methods 2

1.2. Structure of $L_{A,}$ L_B and CGA-1. 2

1.3. Synthesis of ligands. 2

1.3.1 Characterization of L_{A1} 5

1.4. Synthesis of $[Pd_2(L_{A1})_2(L_B)_2](BF_4)_4$ (CGA-3) and $[Pd_2(L_{A1})_4](BF_4)_4$ (C1) 9

2. Dynamic light scattering (DLS) studies 10

2.1. Methods 10

3. TEM under cryo-conditions 10

3.1. Methods 10

3.2. Cryo-TEM images 11

4. In situ liquid cell TEM (LCTEM) study of CGA-3 vesicles in DMSO 12

4.1. Methods 12

4.2. LCTEM images of the CGA-3 vesicles 12

5. Oil-in-oil emulsification 13

5.1. Methods 13

6. Comparison of solubility of the of the surfactants in hexadecane 13

7. Preparation of metal oxide microcapsules from alkoxide precursor containing emulsions. 13

8. DLS studies on emulsion containing Ti(IV) ethoxide 14

9. TEM, STEM studies and EDS elemental mapping of mapping of solid metal oxide microcapsules 15

9.1. Methods. 15

9.2. TEM images of metal oxide capsules. 15

10. Visualization of formation of metal oxide microcapsules using in situ LCTEM technique. 16

11. Stability of the emulsion. 17

12. Formation of metal oxide microcapsules (as floc) 18

13. References 18

1. Experimental Section

1.1. Materials and methods

Unless otherwise stated, all chemicals were obtained from commercial sources and used as received. Compound **L$_B$** was prepared according to literature procedures.[1] GPC purification of all ligands (**L$_{A1}$**, **L$_B$** and their precursors) was performed on a JASCO LC-9210 II NEXT system. Dynamic light scattering experiments were performed on a Malvern Zetasizer ZS nano instrument. NMR spectroscopic data was measured on the spectrometers Bruker AV 500 Avance NEO, AV 600 Avance III HD, AV 700 Avance III HD. For ^{1}H and ^{13}C NMR spectra, chemical shifts were calibrated to the solvent lock signal. All spectra were recorded in standard 5 mm NMR tubes at 25 °C, if not mentioned otherwise. Mass spectrometry and trapped ion mobility data were measured on Bruker ESI-timsTOF (electrospray ionization-trapped ion mobility-time of flight) and Bruker compact high-resolution LC mass spectrometers. For calibration of the TIMS and TOF devices, Agilent ESI-Low Concentration Tuning Mix was used. As lyophilizer, model Alpha 2-4 LSC basic from Christ was used.

1.2. Structure of L$_A$, L$_B$ and CGA-1.

Synthetic details for **L$_A$** and **CGA-1** have been described a previous contribution from our group.[1]

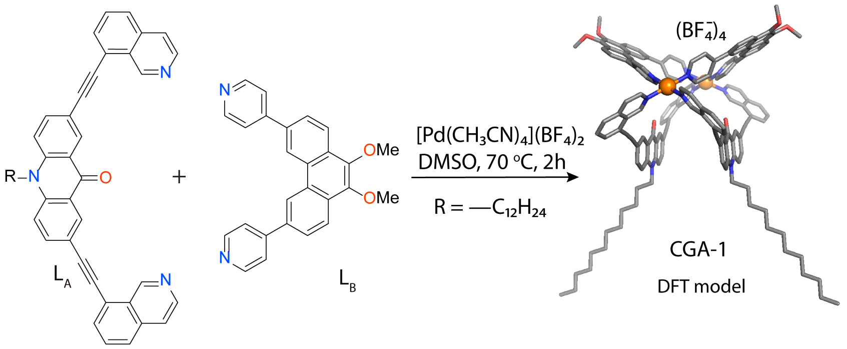

1.3. Synthesis of ligands.

L$_B$ was synthesized by following a procedure reported in a previous contribution from our group.[2]

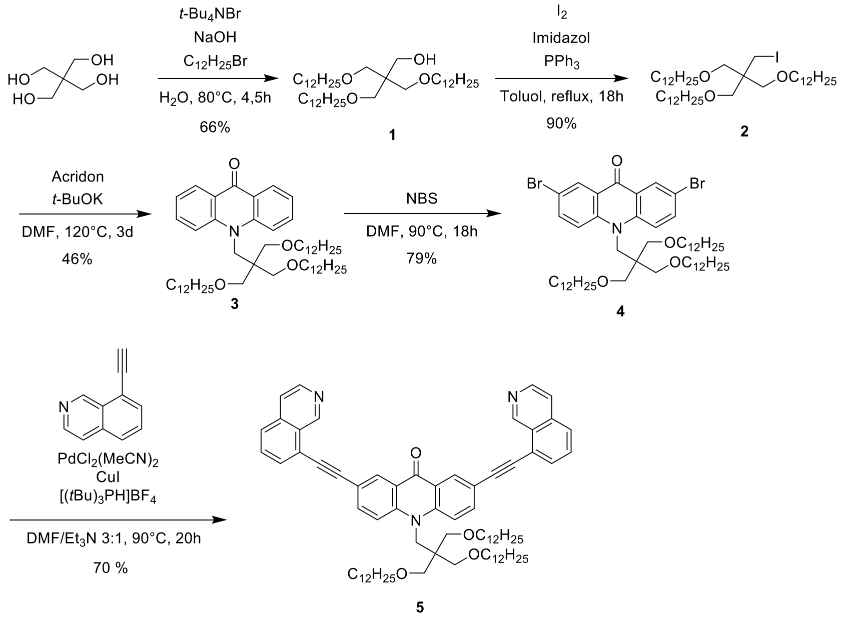

Synthesis of **L$_{A1}$**: Compound 2 was synthesized by following a literature reported procedure.[3]

Synthesis of compound 3

Acridone (103.9 mg, 532.2 µmol, 1.00 eq.) and *t*-BuOK (86.5 mg, 771.0 µmol, 1.45 eq.) were dissolved in DMF (1 mL) and stirred for 1.5 h. **2** (886.9 mg, 1.18 mmol, 2.22 eq.) was added to the reaction mixture and stirred for 3 d at 120 °C. Once allowed to cool, EtOAc (100 mL) was added and the organic layer was washed with saturated NaCl solution (3x50 mL). The solvent was evaporated under reduced pressure and the residue was purified by column chromatography (Isolera Biotage flash chromatography system, *n*-pentane → *n*-pentane/EtOAc 20:1) to yield compound **3** (201.3 mg, 246.0 µmol, 46 %) as a colourless oil.

^{1}H NMR (CD_2Cl_2, 298,15 K, 500 MHz): δ (ppm) = 8.49 (dd, ^3J = 8.0 Hz, ^4J = 1.7 Hz, 2H, H_a), 8.13 (d, ^3J = 8.9 Hz, 2H, H_b), 7.68 (ddd, ^3J = 8.7, ^3J = 6.9, ^4J = 1.7 Hz, 2H, H_c), 7.28 (t, ^3J = 7.4 Hz, 2H, H_d), 4.86 (s, 2H, H (N*CH*$_2$C)), 3.36 (s, 6H, H (C*CH*$_2$OCH$_2$)), 3.25 (t, ^3J = 6.4 Hz, 6H, H (O*CH*$_2$CH$_2$)), 1.50 (p, ^3J = 6.5 Hz, 6H, H (OCH$_2$*CH*$_2$)), 1.27 (s, 54H, H (OCH$_2$CH$_2$(*CH*$_2$)$_9$CH$_3$)), 0.88 (t, ^3J = 6.9 Hz, 9H, H (CH$_2$*CH*$_3$)).

^{13}C NMR (126 MHz, CD_2Cl_2, 298,15 K, 500 MHz): δ (ppm) = 178.13, 144.21, 133.58, 127.64, 122.94, 121.53, 116.90, 71.91, 47.15, 43.36, 32.49, 30.22, 30.05, 26.86, 23.25, 14.45.

IR (ATR): $\tilde{\nu}$ (cm^{-1}) = 2921, 2852, 1737, 1641, 1607, 1488, 1466, 1363, 1259, 1183, 1108, 755, 677.

ESI-HRMS: m/z (%) = 818.70 (100) [M]$^+$, 641.64 (30) [$C_{41}H_{64}NO_3Na$]$^+$

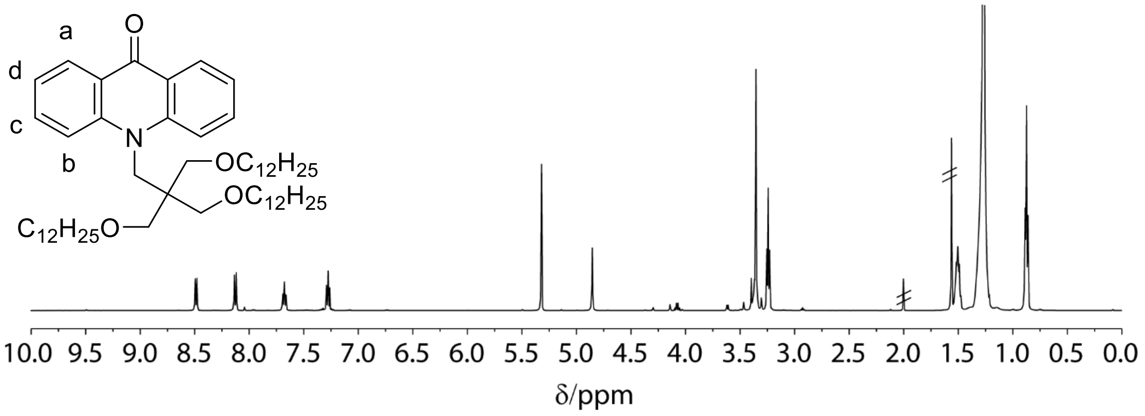

Figure S1. ^{1}H NMR (600 MHz/CDCl$_3$/298 K) of compound **3**.

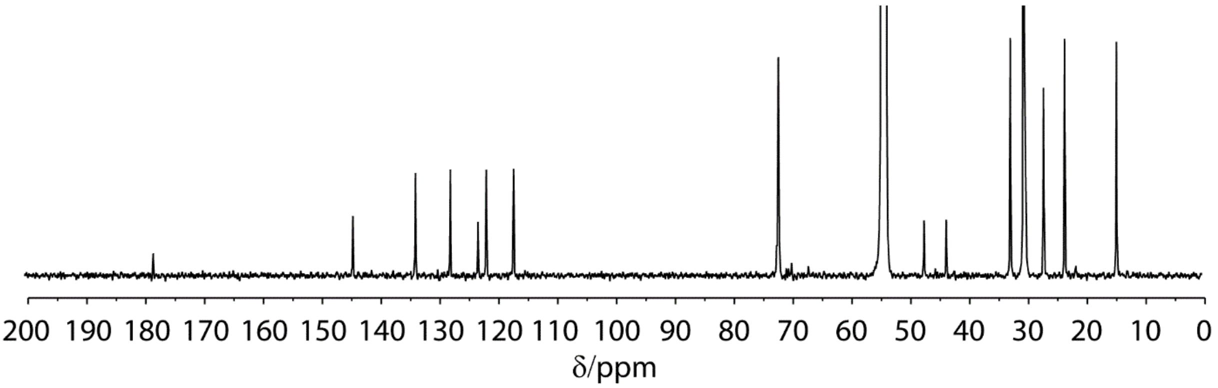

Figure S2. ^{13}C NMR (600 MHz/CDCl$_3$/298 K) of compound **3**.

Synthesis of compound 4

Compound **3** (1.06 g, 1.30 mmol, 1.00 eq.) was dissolved in DMF (5 mL) under N_2-atmosphere. NBS (700.4 mg, 3.94 mmol, 3.04 eq.) was also dissolved in DMF (2 mL) and was slowly added at 0 °C. The mixture was heated to 90 °C for 18 h. The organic phase was extracted with DCM (50 mL) and was washed with saturated NaCl solution (2x100 mL) and cold water (2x100 mL). After evaporation of the solvent under reduced pressure, purification by column chromatography (Isolera Biotage flash chromatography system, *n*-pentane → *n*-pentane/EtOAc 10:1 → *n*-pentane/EtOAc 5:1) yield compound **4** (1.00 g, 1.03 mmol, 79%) as yellow oil.

^{1}H NMR (CD_2Cl_2, 298,15 K, 600 MHz): δ (ppm) = 8.59 (d, ^3J = 2.2 Hz, 2H, H_a), 8.04 (d, J = 9.4 Hz, 2H, H_b), 7.75–7.72 (dd, 2H, H_c), 4.79 (s, 2H, H (N*CH₂*C)), 3.33 (s, 6H, H (CCH_2OCH_2)), 3.23 (t, ^3J = 6.4 Hz, 6H, H (O$CH_2$$CH_2$)), 1.52–1.43 (p, J = 7.3, 6.9 Hz, 6H, H (O$CH_2$$CH_2$)), 1.28 (s, 54H, H (O$CH_2$$CH_2$($CH_2$)$_9$$CH_3$)), 0.92–0.83 (t, ^3J = 6.7 Hz, 9H, H ($CH_2$$CH_3$)).

^{13}C NMR (151 MHz, CD_2Cl_2, 298,15 K): δ (ppm) = 175.92, 142.97, 136.55, 130.02, 124.28, 119.35, 115.27, 72.04, 72.01, 54.36, 54.18, 54.00, 53.82, 53.72, 53.64, 47.13, 43.94, 32.51, 30.29, 30.25, 30.22, 30.18, 30.07, 30.01, 29.95, 26.87, 23.27, 14.56, 14.45.

IR (ATR): $\tilde{\nu}$ (cm^{-1}) = 467, 545, 570, 704, 721, 800, 830, 1021, 1108, 1189, 1256, 1275, 1348, 1470, 1488, 1607, 1643, 2212, 2850, 2919.

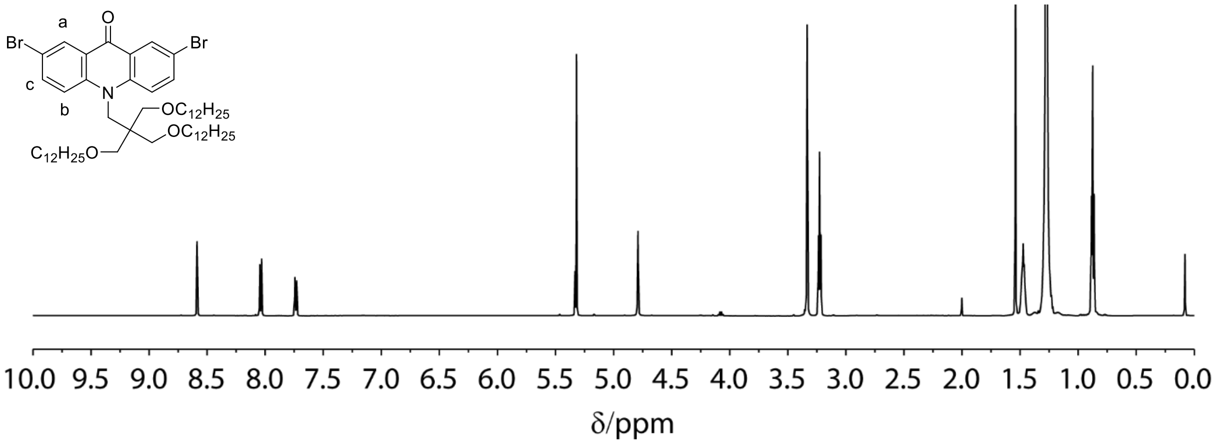

Figure S3. ^{1}H NMR (600 MHz/$CDCl_3$/298 K) of compound **4**.

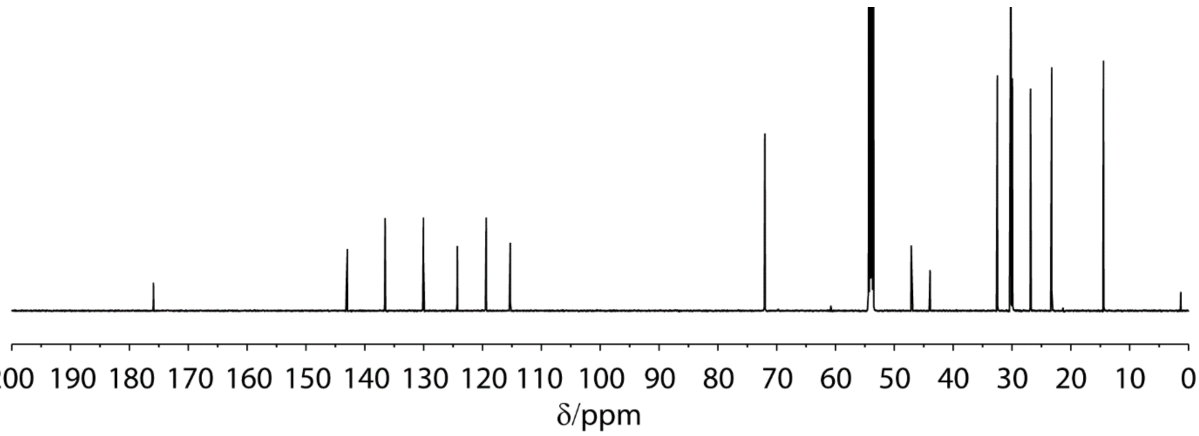

Figure S4. ^{13}C NMR (600 MHz/$CDCl_3$/298 K) of compound **4**.

Synthesis of compound 5 (L_{A1}):

Compound **4** (50 mg, 51.22 µmol, 1.00 eq), 8-ethynylisoquinoline (22.6 mg, 147.5 µmol, 2.88 eq), $PdCl_2(MeCN)_2$ (1 mg, 3.85 µmol, 0.075 eq), CuI (2.30 mg, 12.08 µmol, 0.24 eq), [(*t*Bu)$_3$PH]BF_4 (3.85 mg, 13.27 µmol, 0.26 eq) were dissolved in DMF/Et_3N 3:1 in a Schlenk vessel. Once the solvent was degassed (*via* freeze-thaw cycles) the mixture was subsequently heated at 120°C for 3 days. Once allowed to cool, the mixture was concentrated at reduced pressure and water was added. The organic layer was extracted with DCM. Purification by column chromatography (*n*-pentane/EtOAc 1:1 and then by GPC, yielded the title compound **5** as a yellow solid (39.2 mg, 35.0 µmol, 70 %).

ESI-HRMS: *m/z* = 1121.78 $[M+H]^+$.

1.3.1 Characterization of L_{A1}

^{1}H NMR ($CDCl_3$, 298 K, 600 MHz): *δ* (ppm) = δ 9.89 (s, 2H, H_i), 8.89 (d, *J* = 2.1 Hz, 2H, H_a), 8.62 (d, *J* = 5.5 Hz, 2H, H_h), 8.19 (d, *J* = 9.1 Hz, 2H, H_b), 7.92 (dd, *J* = 6.8 Hz 2H, H_e), 7.89 (d, *J* = 7.2 Hz 2H, H_c), 7.84 (d, *J* = 8.3 Hz 2H, H_f), 7.74 - 7.70 (m, 4H, H_g and H_d), 4.92 (s, 2H, H_j), 3.41 (s, 6H, H_k), 3.29 (t, *J* = 6.5 Hz, 6H, H_l), 1.57 - 1.51 (m, 6H, H_m), 1.31 - 1.19 (m, 54H, H (OC_2H_4-(CH_2)$_9$-CH_3)), 0.83 (t, *J* = 7.0 Hz, 9H, H (CH_2CH_3)).

^{1}H NMR (DMSO-d$_6$, 320 K, 500 MHz): *δ* (ppm) = δ 9.78 (s, 2H, H_i), 8.67 (d, *J* = 2.1 Hz, 2H, H_a), 8.63 (d, *J* = 5.5 Hz, 2H, H_h), 8.21 (d, *J* = 9.1 Hz, 2H, H_b), 8.05- 8.02 (m, 4H, H_d and H_c), 7.97 (d, *J* = 7.2 Hz 2H, H_f), 7.90 (d, *J* = 5.72 Hz 2H, H_g), 7.83 (t, *J* = 7.75 Hz, 2H, H_e), 4.87 (s, 2H, H_j), 3.40 (s, 6H, H_k), 3.21 (t, *J* = 6.5 Hz, 6H, H_l), 1.41 (t, *J* = 5.6 Hz, 6H, H_m), 1.25 - 1.15 (m, 54H, H (OC_2H_4-(CH_2)$_9$-CH_3)), 0.79 (t, *J* = 6.5 Hz, 9H, H (CH_2CH_3)).

^{13}C NMR (150 MHz, $CDCl_3$, 298 K): *δ* (ppm) = 176.73, 151.02, 143.39, 135.96, 135.91, 131.67, 131.58, 130.14, 127.94,126.91, 122.56, 121.92, 120.74, 117.01, 115.93, 95.39, 85.92, 71.58, 46.69, 43.30, 31.91, 29.73, 29.71, 29.68, 29.56, 29.36, 26.35, 22.67, 14.10.

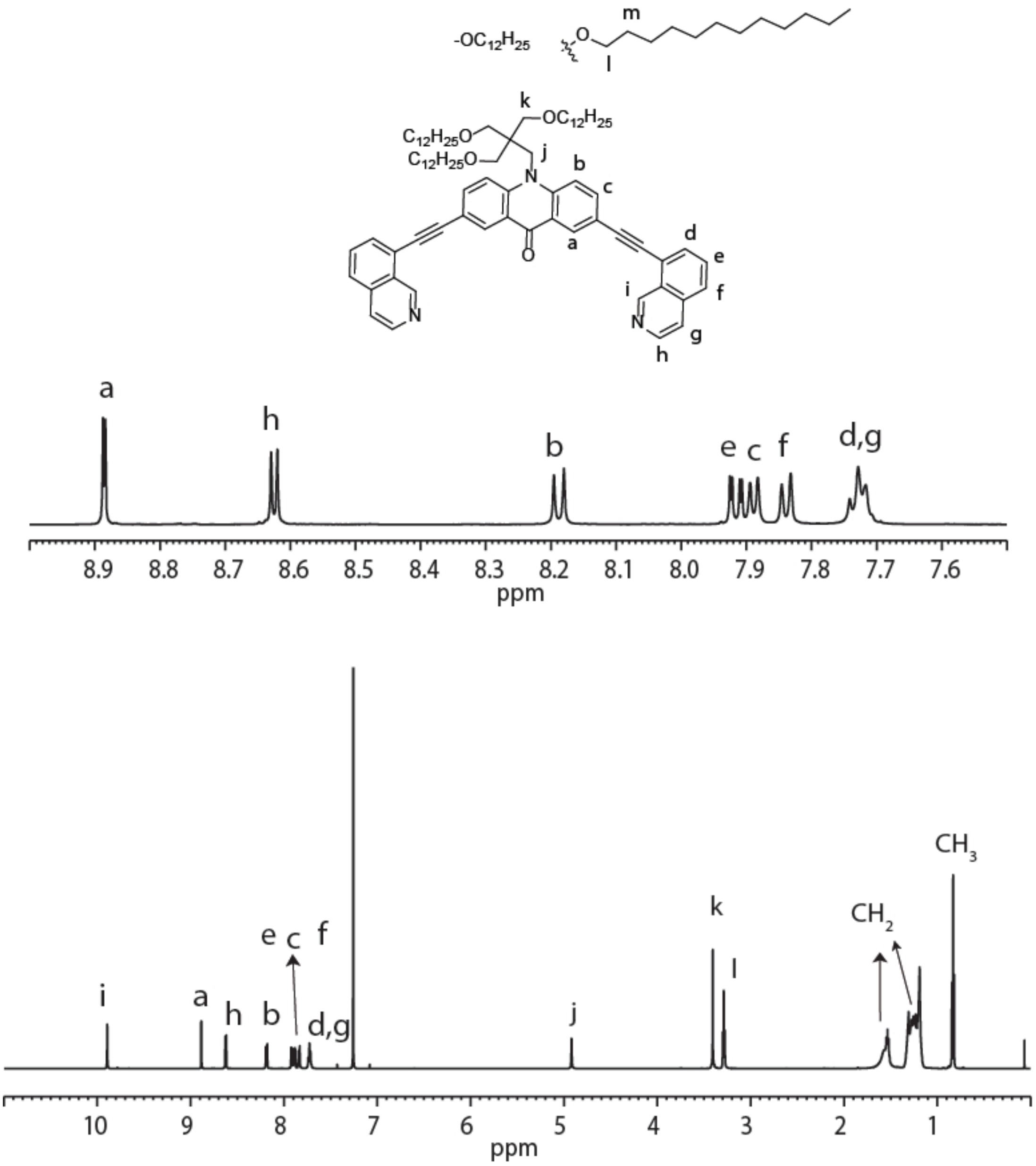

Figure S5. ^{1}H NMR (600 MHz/$CDCl_3$/298 K) of $\mathbf{L_{A1}}$ (compound **5**).

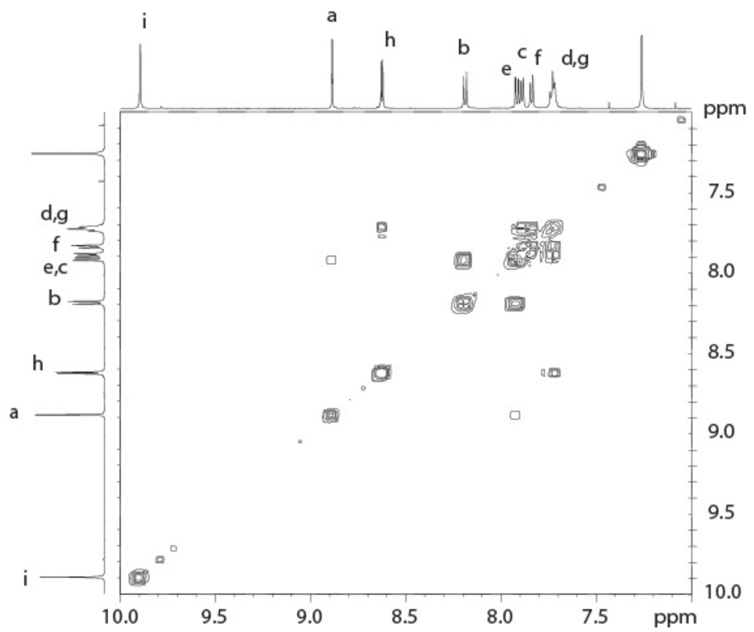

Figure S6. ^{1}H – ^{1}H COSY spectrum aromatic region (500 MHz/ $CDCl_3$/298 K) of $\mathbf{L_{A1}}$ (compound **5**).

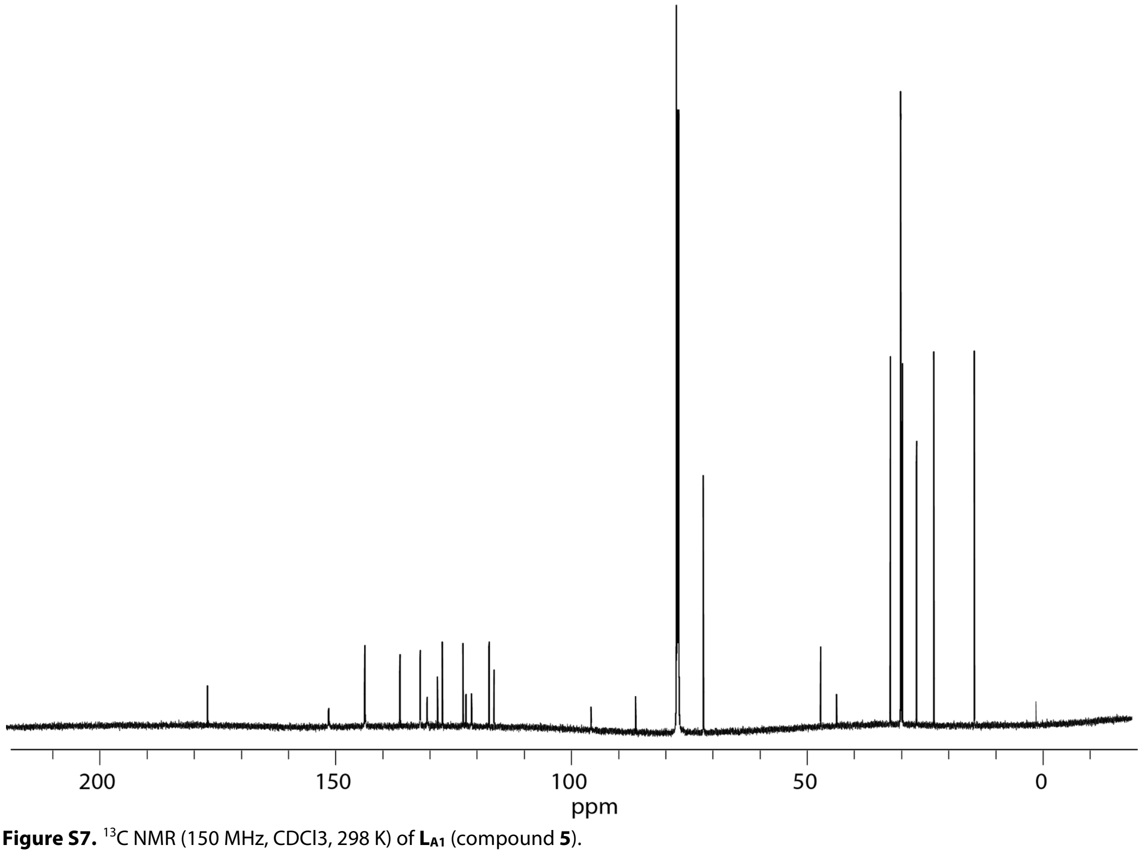

Figure S7. ^{13}C NMR (150 MHz, CDCl3, 298 K) of $\mathbf{L_{A1}}$ (compound **5**).

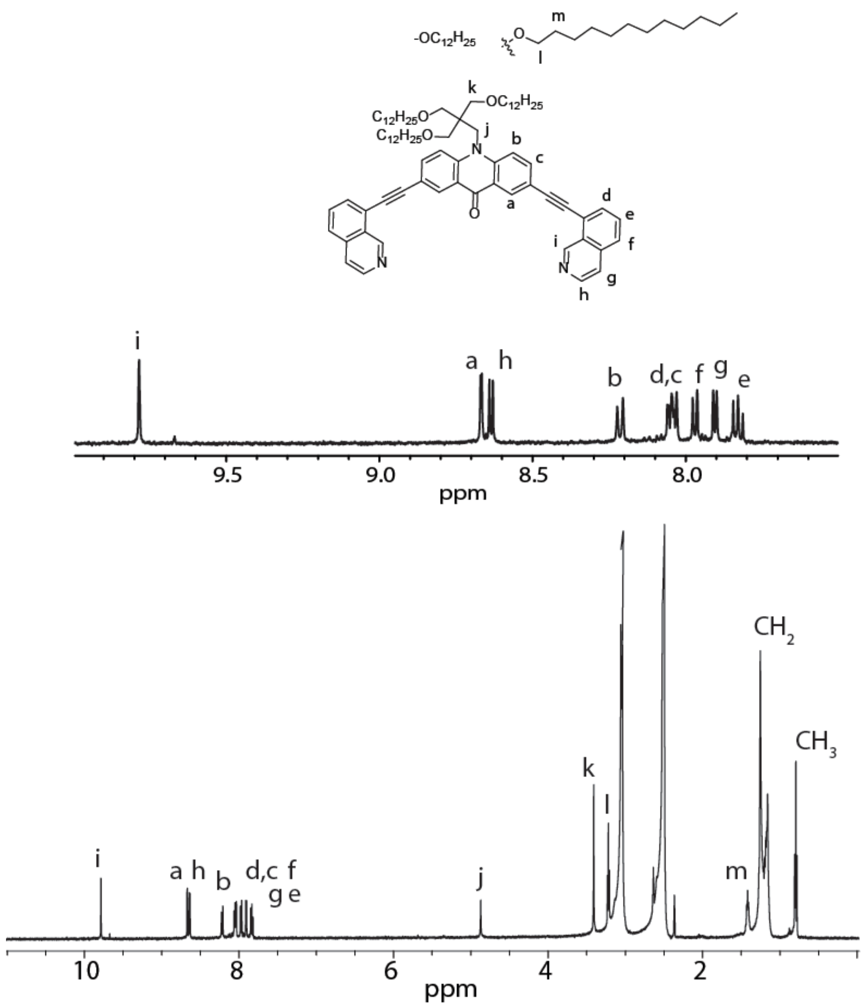

Figure S8. ^{1}H NMR (500 MHz/DMSO-D6/320 K) of $\mathbf{L_{A1}}$ (compound **5**).

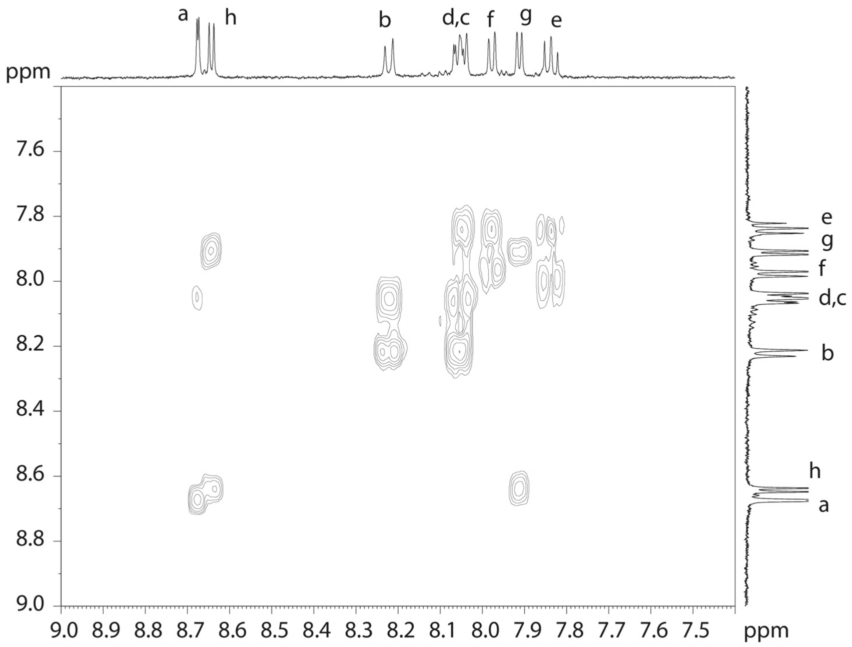

Figure S9. ^{1}H – ^{1}H COSY spectrum aromatic region (500 MHz/DMSO-D6/320 K) of $\mathbf{L_{A1}}$ (compound **5**).

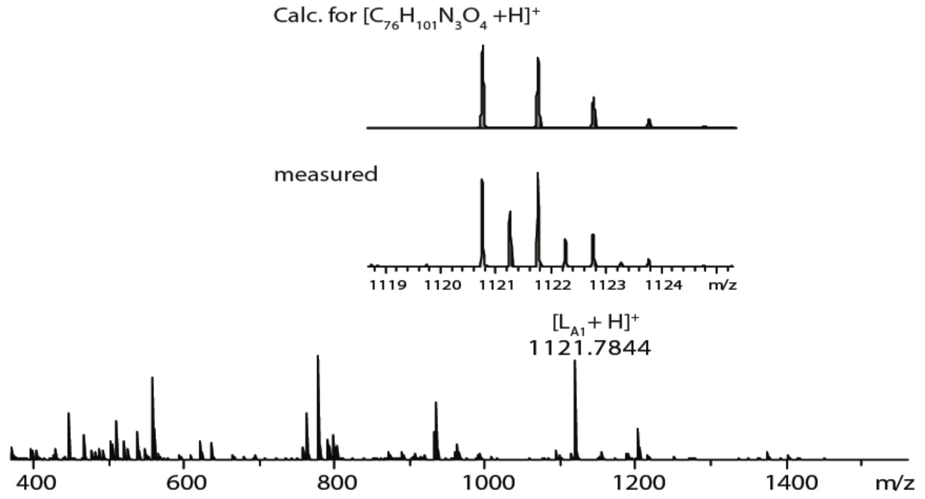

Figure S10. ESI mass spectrum of ligand $\mathbf{L_{A1}}$ (compound **5**).

1.4. Synthesis of $[Pd_2(L_{A1})_2(L_B)_2](BF_4)_4$ (**CGA-3**) and $[Pd_2(L_{A1})_4](BF_4)_4$ (**C1**)

CGA3: A solution of $[Pd(CH_3CN)_4](BF_4)_2$ (60 µL, 15 mM/DMSO-*d*$_6$, 0.9 µmol) was combined with a suspension of $\mathbf{L_{A1}}$ (120 µL, 7 mM/ DMSO-*d*$_6$, 0.84 µmol) and a solution of $\mathbf{L_B}$ (120 µL, 7 mM/ DMSO-*d*$_6$, 0.84 µmol) in DMSO-*d*$_6$ (300 µL) and heated at 80 °C for 96 h to afford $[Pd_2(\mathbf{L_{A1}})_2(\mathbf{L_B})_2](BF_4)_4$ (**CGA-3**).

C1: A solution of $[Pd(CH_3CN)_4](BF_4)_2$ (60 µL, 15 mM/DMSO-*d*$_6$, 0.9 µmol) was combined with a suspension of $\mathbf{L_{A1}}$ (120 µL, 15 mM/ DMSO-*d*$_6$, 1.68 µmol) in DMSO-*d*$_6$ (300 µL) and heated at 80 °C for 96 h to afford $[Pd_2(\mathbf{L_{A1}})_4](BF_4)_4$ (**C1**).

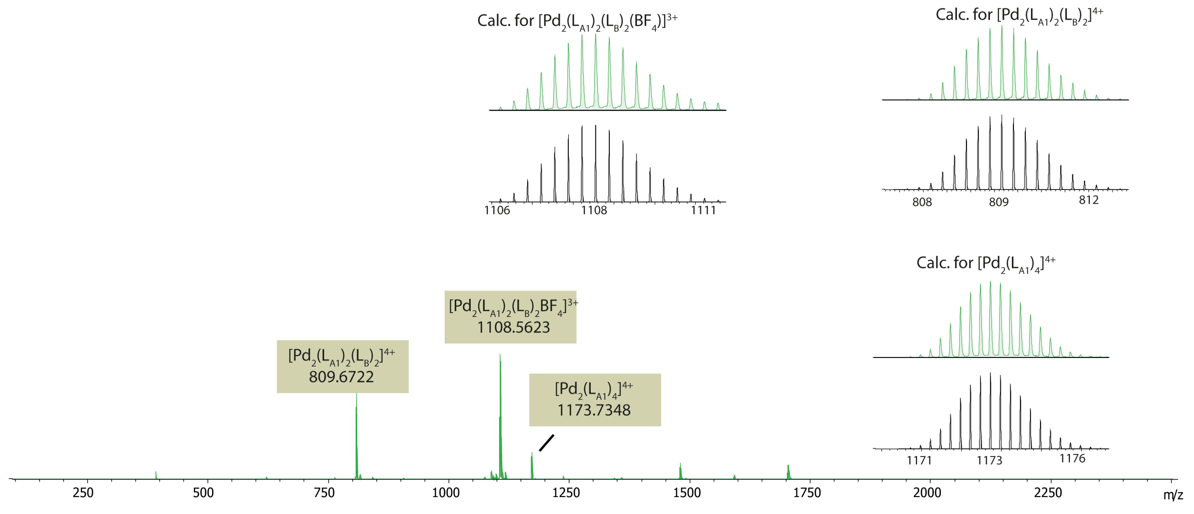

Figure S11. ESI mass spectrum of **CGA-3**. The measured and calculated isotope pattern for **CGA-3** is shown in the inset.

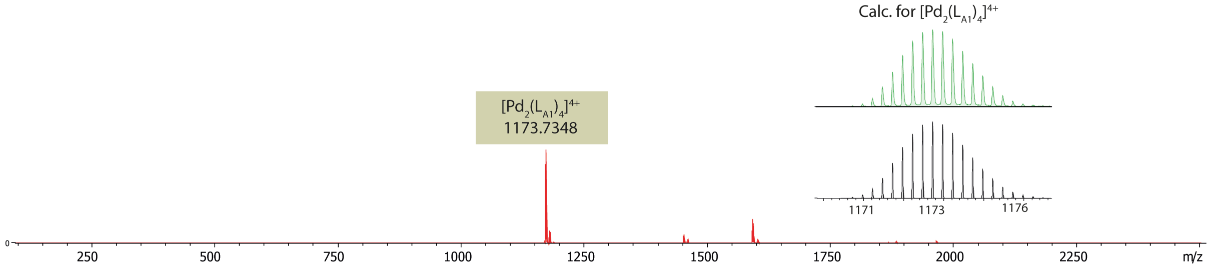

Figure S12. ESI mass spectrum of homoleptic cage **C1**. The measured and calculated isotope pattern for **C1** is shown in the inset.

2. Dynamic light scattering (DLS) studies

2.1. Methods

Lyophilized powder of CGA-3 was dissolved in DMSO (concentrations 0.2mM and 0.1mM). These solutions were characterized by DLS to determine the hydrodynamic diameter of the colloidal particles.

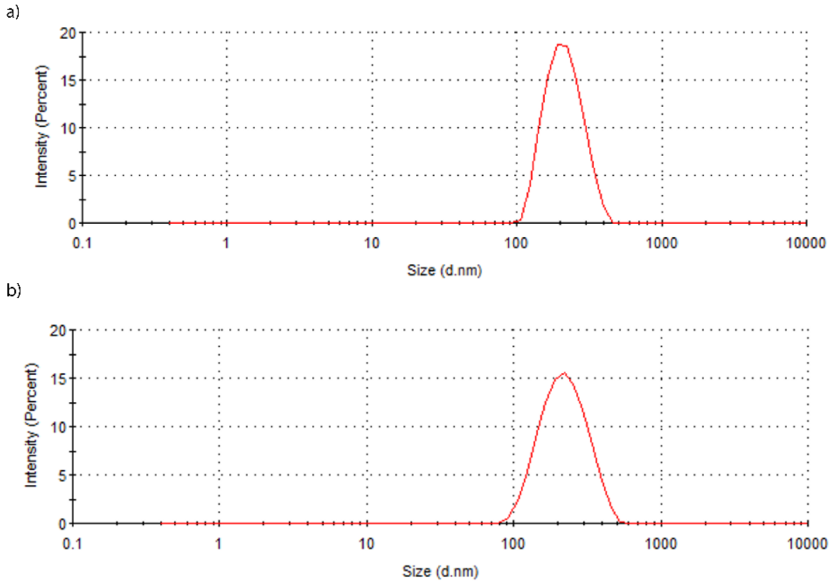

Figure S13. Dynamic light scattering reveals that the hydrodynamic diameters of the vesicles are in the range of i) 190 nm (concentration = 0.2 mM; PDI = 0.134) and ii) 220 nm (concentration = 0.1 mM; PDI = 0.152).

3. TEM under cryo-conditions

3.1. Methods

4µl of sample in 0.2mM DMSO were applied to a glow-discharged holey carbon grid (Quanifoil 2/1, Quantifoil). The sample was than blotted for 3 seconds and plunged into liquid ethane using a Cryoplunge 3 with GentleBlot (Gatan). Digital micrographs have been recorded on a FEI Talos Arctica electron microscope operating at 200kV using a Falcon III (FEI) direct electron detector. Images were acquired at a nominal magnification of 25,000x with a pixel size of 0.372nm.

3.2. Cryo-TEM images

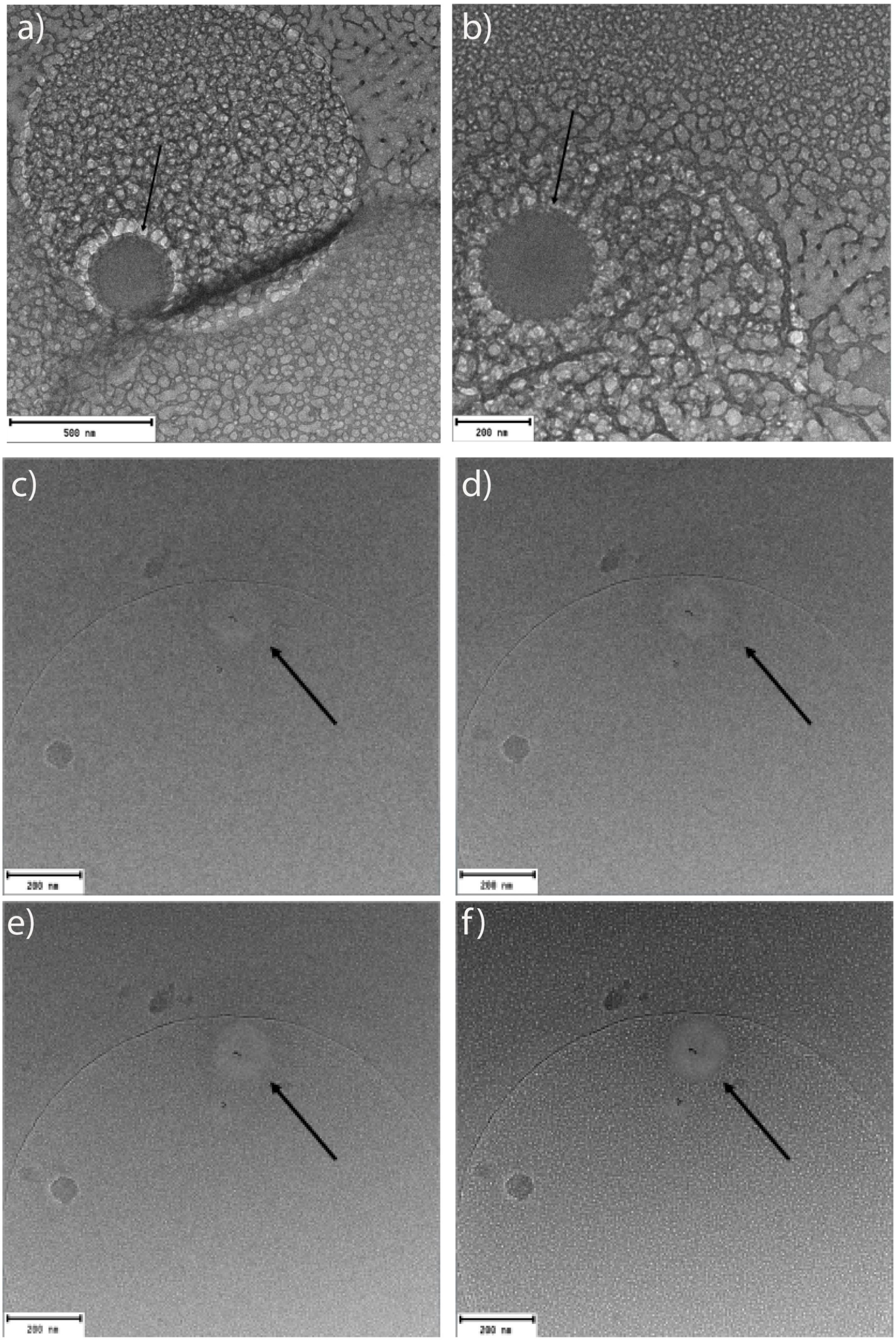

Figure S14. Representative cryoEM micrographs of CGA-3-vesicles in 0.2mM DMSO. (a-b) CryoEM images of CGA- 3-vesicles in 0.2mM DMSO recorded with a relatively high total electron dose of 300e-/Å2. Scale bars 500nm and 200nm. (c-d) Consecutive cryoEM images of CGA-3-vesicles in 0.2mM DMSO recorded at the same position. Each image was recorded with a total exposure of 60e-/Å2. Scale bar, 200nm. Black arrows indicate the CGA-3 vesicles.

4. In situ liquid cell TEM (LCTEM) study of CGA-3 vesicles in DMSO

4.1. Methods

STEM imaging was performed using a JEOL microscope (JEM-2800) with a Schottky-type emission source working at 200 kV (energy spread of 0.75 eV), equipped with a Gatan OneView camera (4k x 4k, 25FPS). The spherical aberration (Cs) of the objective lens is 0.7 mm, and the chromatic aberration (Cc) is 1.3 mm, which results in a point-to-point resolution of 0.21 nm. Energy dispersive spectroscopy (EDS) mapping was performed with the equipped double SDD detectors, with a solid angle of 0.98 steradians with a detection area of 100 mm^2.

The experiment of *in situ* liquid TEM was carried out in the JEM-2800 microscope mentioned above, with the electrochemistry liquid holder Poseidon 210 (Protochips Inc) on the E-chips. The window of the observable area has a size of 55x55 μm, sandwiched by two silicon nitride membrane of 50 nm thickness. The liquid thickness was 150 nm, presumably defined by the spacer. The probe size is 0.3 nm in STEM mode with an optimized scan rate to provide a suitable electron dose, yet not to affect the sample or create any bubble formation during the observation.

4.2. LCTEM images of the CGA-3 vesicles

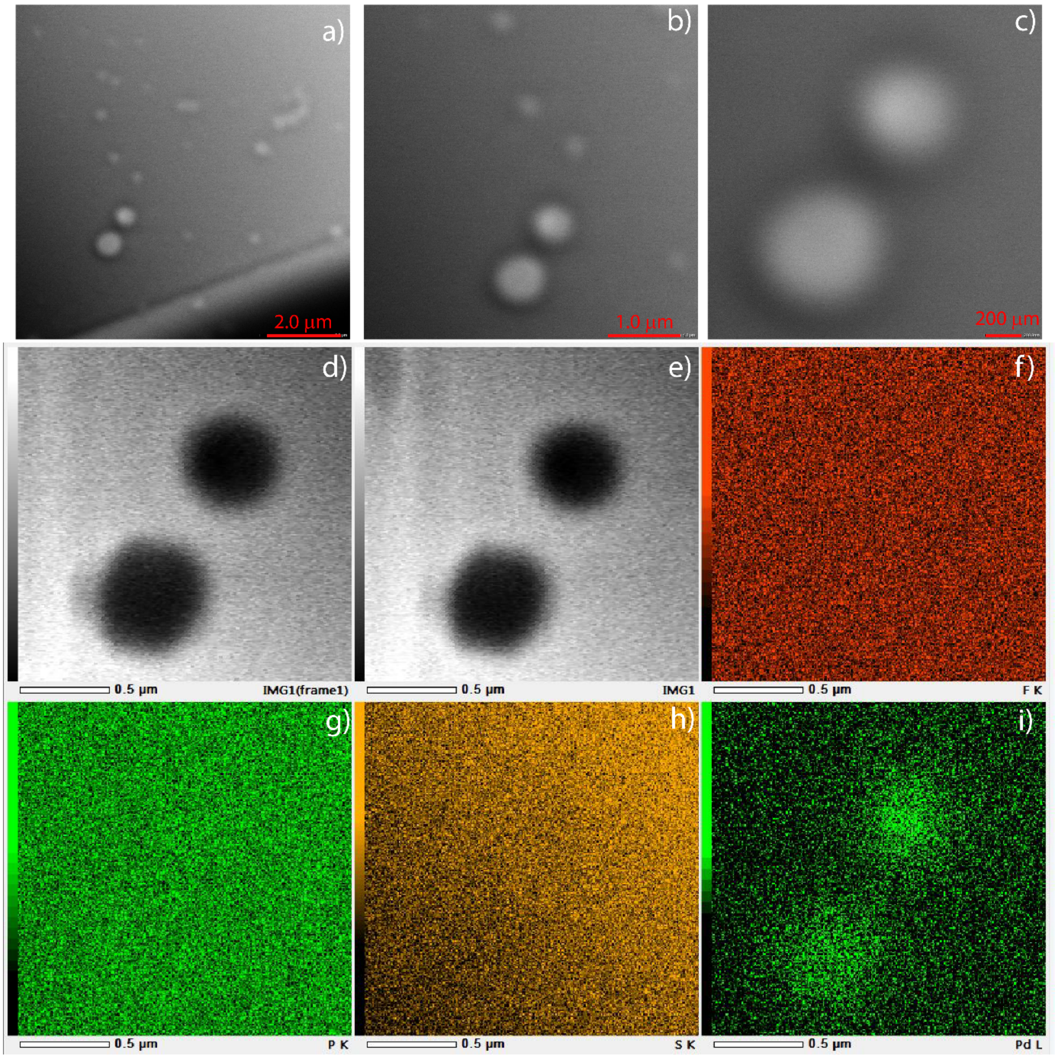

Figure S15. ***In situ* STEM imaging**. a-c) STEM–high-angle annular dark-field microscopy (HAADF) images of CGA-3 vesicles in DMSO within the *in situ* liquid stage; d) and e) are STEM-BF (bright field) images of the vesicles before and after EDS elementary intensity analysis mapping experiment, respectively; f-g) EDS elementary intensity analysis mapping shows the vesicles are made up of Pd-based compounds.

5. Oil-in-oil emulsification

5.1. Methods

All experiments were done in 1 ml mixtures of DMSO (containing lyophilized solid CGA-3 as emulsifier) and hexadecane (HD) in the ratio 4:1. The mixtures were subjected to homogenization by vortexing for two minutes.

6. Comparison of solubility of the of the surfactants in hexadecane

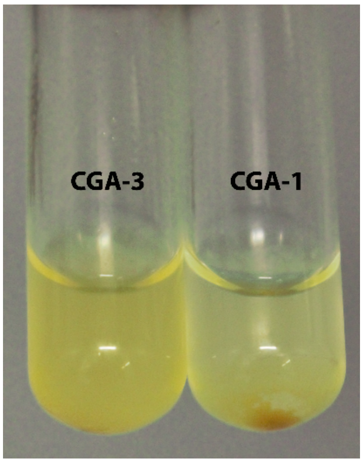

Figure S16. Comparative solubility study of CGA-3 and CGA-1 (0.05mM in hexadecane) after 30 minutes of dissolution.

7. Preparation of metal oxide microcapsules from alkoxide precursor-containing emulsions

Titanium oxide:

Formation of titanium oxide microcapsules from emulsion (R1T): Emulsions were formed using 0.56 mg CGA-3 dissolved in 200 mg dry DMSO and 20 mg dry hexadecane solution containing 1 mg $Ti(OEt)_4$ (emulsion T1). This mixture was vortexed for 2 min and 100μl of DMSO containing 1μl of distilled water was added to initiate the hydrolysis reaction. The water to alkoxide molar ratio was typically 4 : 1. Below this ratio, the reaction did not complete. Centrifuging and rinsing with ethanol were repeatedly applied to remove the solvents and surfactant to give a product that was subsequently dried under vacuum.

Formation of titanium oxide microcapsules from emulsion (R2T): Normal emulsions were formed using 0.56 mg CGA-3 dissolved in 200 mg dry DMSO and 20 mg dry hexadecane solution containing 2 mg $Ti(OEt)_4$ (emulsion T2). This mixture was vortexed for 2 min and 100μl of DMSO containing 2μl of distilled water was added to initiate the hydrolysis reaction. The water to alkoxide molar ratio was typically 4 : 1. Below this ratio, the reaction did not complete. Centrifuging and rinsing with ethanol were repeatedly applied to remove the solvents and surfactant to give a product that was subsequently dried under vacuum.

Zirconium oxide:

Formation of zirconium oxide microcapsules from emulsion (R1Z): Normal emulsions were formed using 0.56 mg CGA-3 dissolved in 200 mg dry DMSO and 20 mg dry hexadecane solution containing 2.76 mg of (80% $Zr(OBu)_4$ in n-butanol) (emulsion Z1). This mixture was vortexed for 2 min and 100μl of DMSO containing 1μl of distilled water was added to initiate the hydrolysis reaction. The water to alkoxide molar ratio was typically 4 : 1. Below this ratio, the reaction did not complete. Centrifuging and rinsing with ethanol were repeatedly applied to remove the solvents and surfactant to give a product that was subsequently dried under vacuum.

Formation of zirconium oxide microcapsules from emulsion (R2Z): Normal emulsions were formed using 0.56 mg CGA-3 dissolved in 200 mg dry DMSO and 20 mg dry hexadecane solution containing 5.52 mg of (80% $Zr(OBu)_4$ in *n*-butanol) (emulsion Z2). This mixture was vortexed for 2 min and 100μl of DMSO containing 2μl of distilled water was added to initiate the hydrolysis reaction. The water to alkoxide molar ratio was typically 4 : 1. Below this ratio, the reaction did not complete. Centrifuging and rinsing with ethanol were repeatedly applied to remove the solvents and surfactant to give a product that was subsequently dried under vacuum.

Niobium oxide:

Formation of titanium oxide microcapsules from emulsion (R1N): Normal emulsions were formed using 0.56 mg CGA-3 dissolved in 200 mg dry DMSO and 20 mg dry hexadecane solution containing 2.015 mg $Nb(OBu)_5$ (emulsion N1). This mixture was vortexed for 2 min and 100μl of DMSO containing 1μl of distilled water was added to initiate the hydrolysis reaction. The water to alkoxide molar ratio was typically 4 : 1. Below this ratio, the reaction did not complete. Centrifuging and rinsing with ethanol were repeatedly applied to remove the solvents and surfactant to give a product that was subsequently dried under vacuum.

Formation of titanium oxide microcapsules from emulsion (R2N): Normal emulsions were formed using 0.56 mg CGA-3 dissolved in 200 mg dry DMSO and dry 20 mg hexadecane solution containing 4.03 mg $Nb(OBu)_5$ (emulsion N2). This mixture was vortexed for 2 min and 100μl of DMSO containing 2μl of distilled water was added to initiate the hydrolysis reaction. The water to alkoxide molar ratio was typically 4 : 1. Below this ratio, the reaction did not complete. Centrifuging and rinsing with ethanol were repeatedly applied to remove the solvents and surfactant to give a product that was subsequently dried under vacuum.

8. DLS studies on emulsion containing Ti(IV) ethoxide

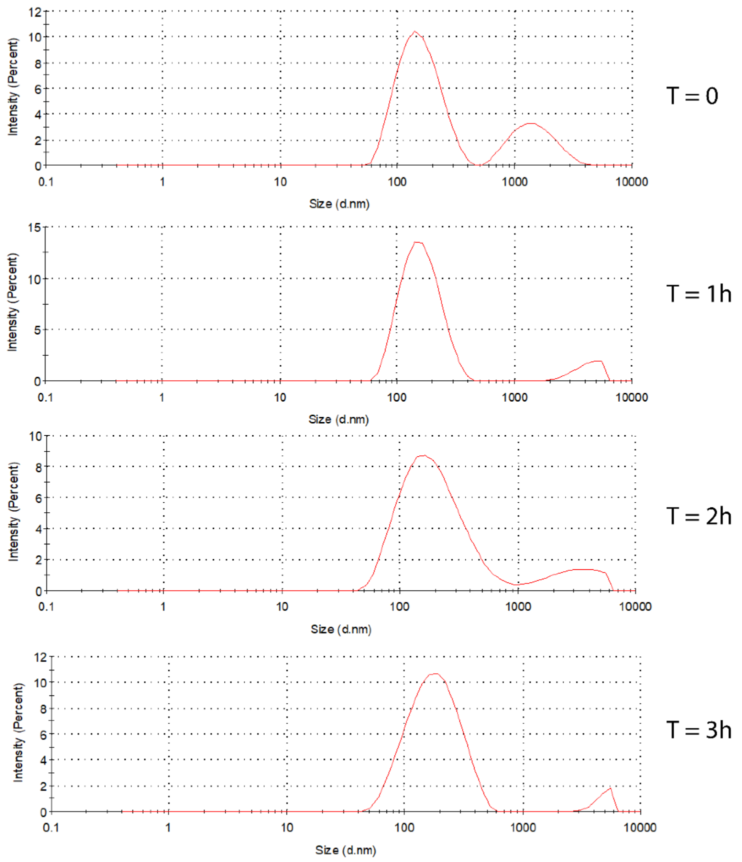

Figure S17. Time-dependent DLS studies of emulsion T1 (experiments were done with minimum exposure to ambient atmosphere to reduce the possibilities of hydrolysis of Ti(IV) ethoxide).

9. TEM, STEM studies and EDS elemental mapping of solid metal oxide microcapsules

9.1. Methods

The same TEM instrument as mentioned in the LCTEM experiment was used to perform the following dry-state imaging tasks. The samples were dropcasted onto a typical commercial copper mesh with carbon support. Afterward the samples were dried overnight in a desiccator before introduction into the TEM column.

9.2. TEM images of metal oxide capsules.

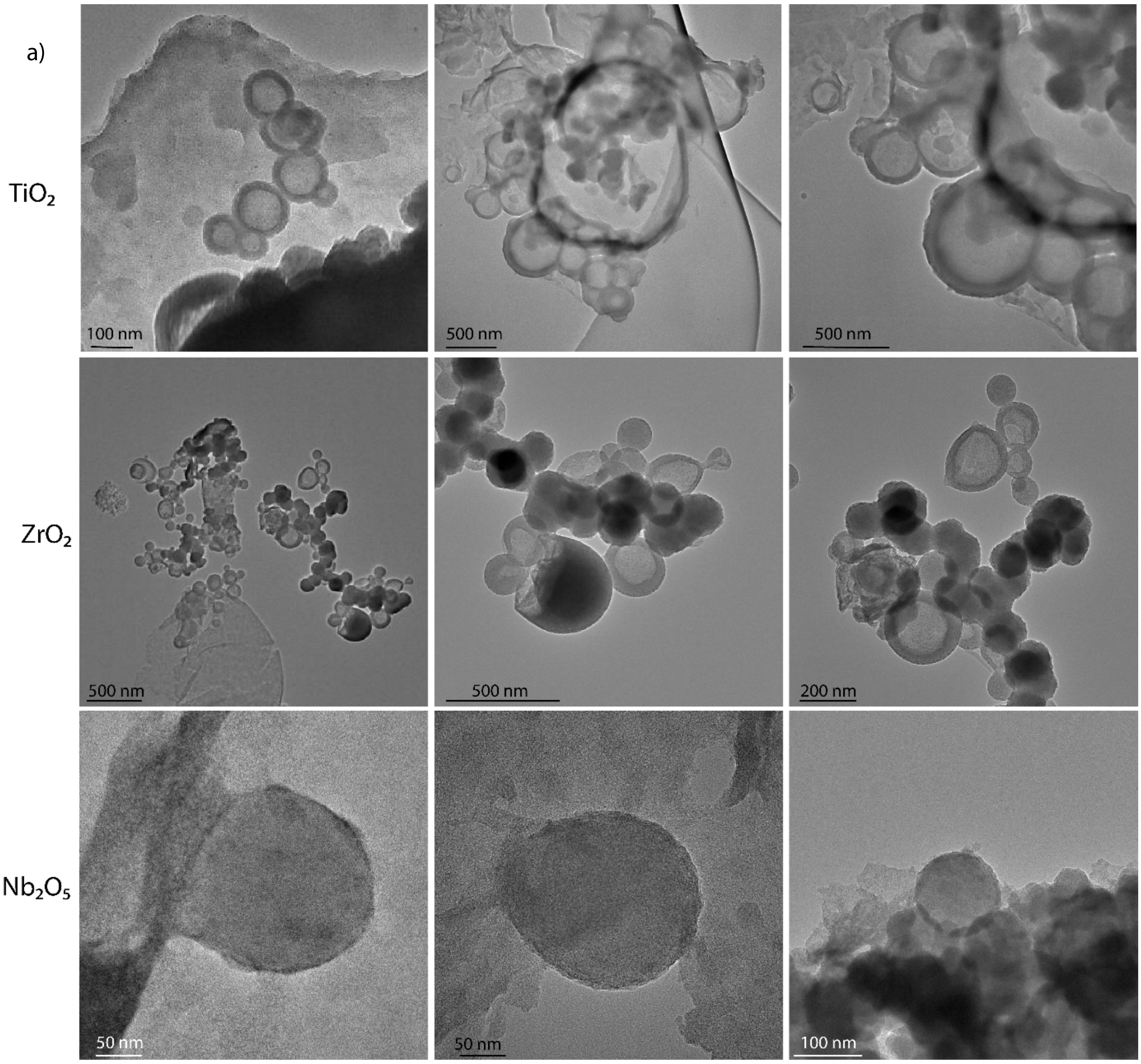

b)

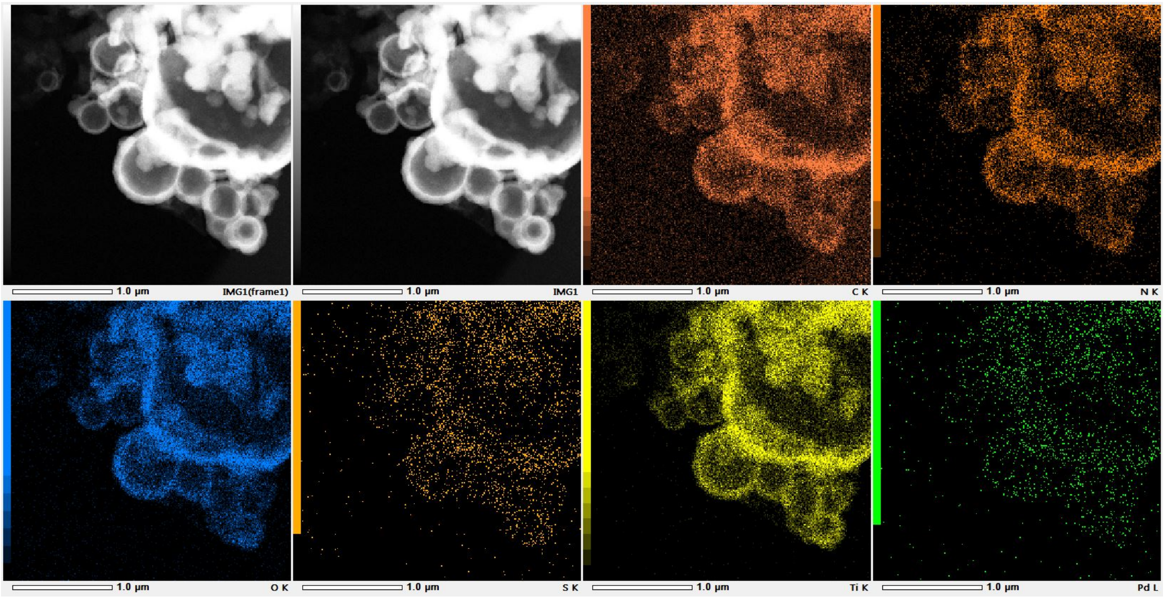

Figure S18. a) TEM images of titanium oxide (top panel), zirconium oxide (middle panel) and niobium oxide (bottom panel); b) EDX elemental intensity maps of the titanium oxide microspheres.

10. Visualization of metal oxide microcapsule formation using in situ LCTEM technique

10.1 Methods

For in situ liquid-cell TEM experiments, we used a Protochips Poseidon Select heating holder (i.e. using 2 separate inlets and 1 outlet). Firstly, the liquid-cell chips (with 50 nm silicon nitride thickness) were cleaned in acetone and methanol to remove the photoresist layer. Subsequently, top and bottom chips were plasma cleaned for 5 minutes to induce hydrophilicity. Then the chips were filled with the emulsion (R1T or R2Z; see section 7). Finally, the chips were assembled in the tip of the holder, and leak-checked to prevent breakage of the column vacuum during the experiment. After inserting the holder into the column of the microscope, the water-DMSO mixture (100μl of DMSO containing 1μl of distilled water) was allowed to flow through the inlet using a syringe pump at the rate of 1μl/min. Before the introduction of water, dense spherical droplets (the dispersed phase hexadecane containing metal oxide precursors) were seen. However, after 15 minutes of the injection of water, the formation of hollow spherical particles (metal oxide microcapsules) emerged from the hexadecane droplets. The below picture showcases real-time imaging of the conversion of two dense spheres to hollow spherical particles. From the images, it is evident that the process takes ~12 s to complete.

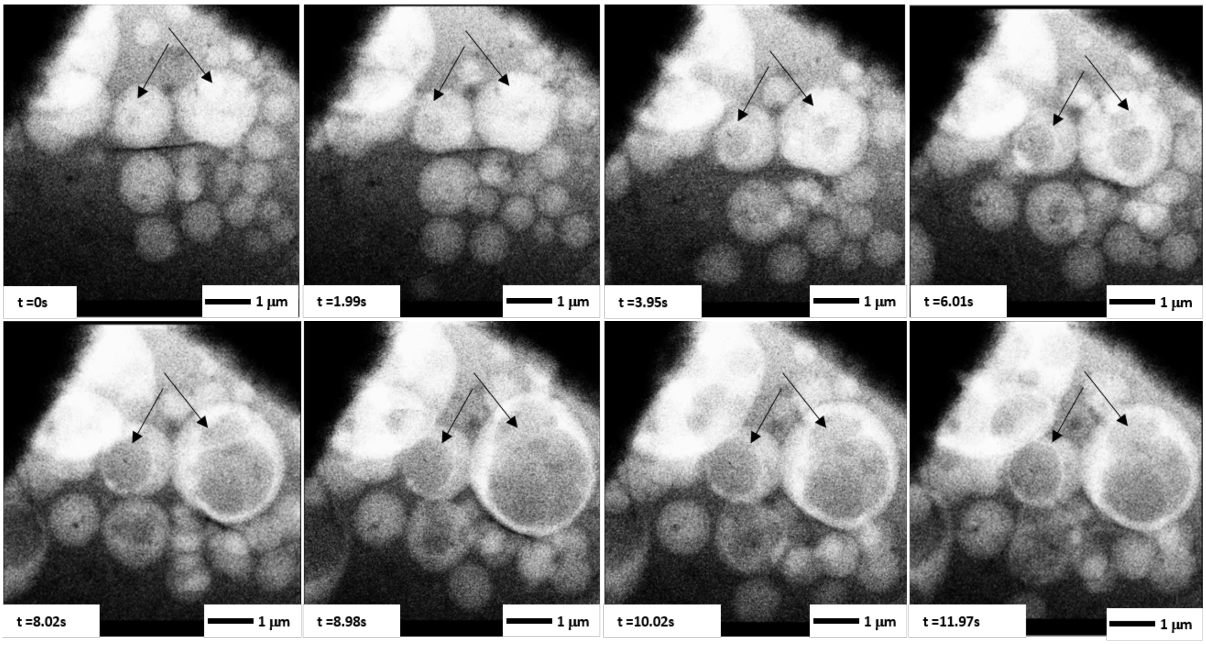

Figure S19. Snapshots of growth of zirconium oxide acquired by LCTEM (STEM-BF imaging).

11. Stability of the emulsion.

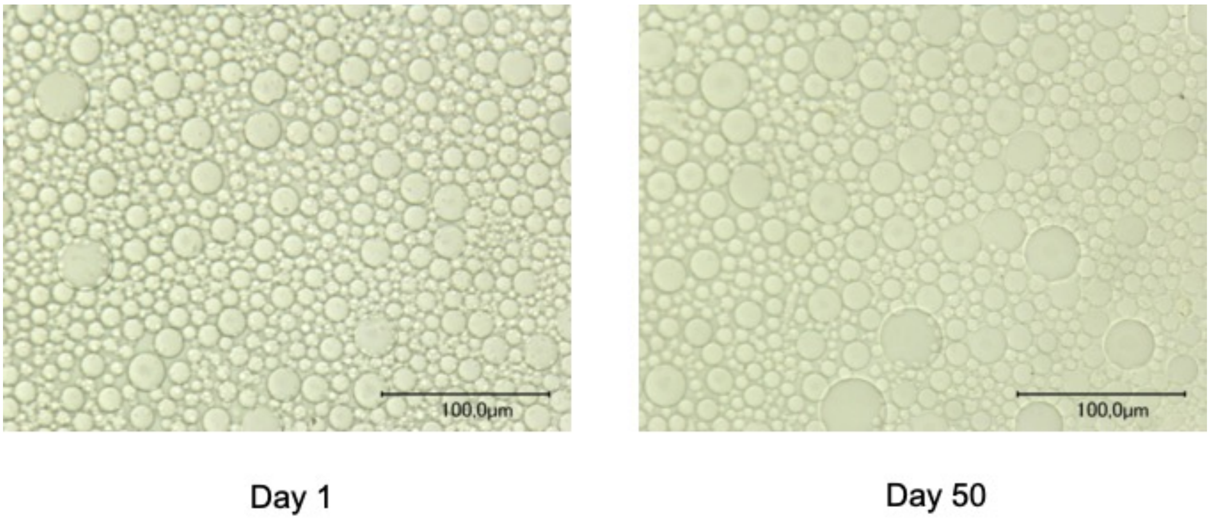

Figure S20. Time-dependent stability check (day 1 and day 50) of typical emulsion (DMSO: HD = 4:1; Surfactant = **CGA-3** (0.2mM) at 25 °C).

12. Formation of metal oxide microcapsules (as flocculation)

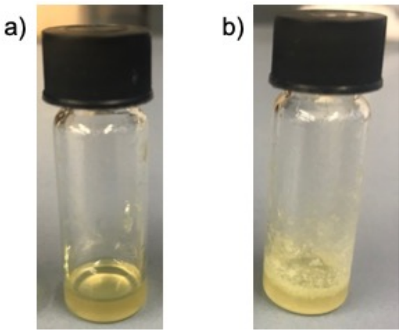

Figure S21. a) The emulsion containing the precursor of titanium oxide (in the dispersed hexdecane phase) before addition of water; b) after hydrolytic conversion of $Ti(OEt)_4$ to titanium oxide microspheres (as flocculation) after addition of water.

13. References.

1. S. Saha, B. Holzapfel, Y.-T. Chen, K. Terlinden, P. Lill, C. Gatsogiannis, H. Rehage, G. H. Clever, *J. Am. Chem. Soc.* **2018**, *140,* 17384-17388.

2. W. M. Bloch, Y. Abe, J. J. Holstein, C. M. Wandtke, B. Dittrich, G. H. Clever, *J. Am. Chem. Soc.* **2016**, *138*, 13750-13755.

3. S.-I. Kawano, Y. Ishida, K. Tanaka, *J. Am. Chem. Soc.* **2015**, *137*, 2295–2302.
